# Electrokinetic properties of healthy and β-thalassemia erythrocyte membranes under *in vitro* exposure to static magnetic field

**DOI:** 10.3389/fchem.2023.1197210

**Published:** 2023-10-19

**Authors:** Virjinia Doltchinkova, Siya Lozanova, Blaga Rukova, Rumin Nikolov, Elitsa Ivanova, Chavdar Roumenin

**Affiliations:** ^1^ Department of Biophysics and Radiobiology, Faculty of Biology, Sofia University “St. Kliment Ohridski”, Sofia, Bulgaria; ^2^ Institute of Robotics “St. Ap. and Gospeller Matthew”, Bulgarian Academy of Sciences, Sofia, Bulgaria; ^3^ Department of Medical Genetics, Medical University of Sofia, Sofia, Bulgaria; ^4^ Faculty of Mechanical Engineering, Technical University, Sofia, Bulgaria

**Keywords:** static magnetic field, zeta potential, erythrocyte membranes, microelectrophoresis, beta-thalassemia, lipid peroxidation, fluorescence microscopy

## Abstract

**Introduction:** The current understanding of the biological impacts of a static magnetic field (SMF) is restricted to the direct interactions of the magnetic field with biological membranes. The electrokinetic (zeta) potential is an electrochemical property of erythrocyte surfaces which was negatively charged in physiological media after SMF exposure (0.1‒2.0 T).

**Methods:** The novel data about electrokinetic parameters of the erythrocytes is determined by microelectrophoresis after SMF-exposure in norm and heterozygous β-thalassemia. The methods of light scattering, lipid peroxidation, fluorescence microscopy are used.

**Results:** The electrokinetic potential of erythrocytes in norm is increased after SMF intensities due to enhanced negatively exposed charges on the outer surface of the membrane accompanied by an increase in light scattering where changes in cell morphology are observed. Conversely, a decrease in the zeta potential of β-thalassemia erythrocytes upon SMF-treatment was determined because of the reduction in the surface electrical charge of the membranes, where a significant decrease in light scattering at 1.5 T and 2.0 T was recorded. Exposure to SMF (0.5–2.0 T) was associated with an increase in the malondialdehyde content in erythrocytes. Biophysical studies regarding the influence of SMF on the electrostatic free energy of cells shows an increase in negative values in healthy erythrocytes, which corresponds to the implementation of a spontaneous process. This is also the process in β-thalassemia cells after SMF exposure with lower negative values of free electrostatic energy than erythrocytes in norm.

**Discussion:** The effect of static magnetic field (SMF 0.1–2.0 T) on the electrokinetic and morphological characteristics of erythrocytes in norm and β-thalassemia is determined and correlated with the increase/reduction in surface charge and shrinkage/swelling of the cells, respectively. Lipid peroxidation of healthy and β-thalassemia erythrocytes caused an enhancement of lipid peroxidation because of the higher concentrations of TBARS products in cellular suspension. SMF (0.1‒2.0 T) altered the spontaneous chemical processes with negative values of electrostatic free energy of erythrocytes in norm and β-thalassemia accompanied by a lower FITC-Concanavalin A binding affinity to membrane receptors (SMF 2.0 T). The electrokinetic properties of human erythrocytes in norm and β-thalassemia upon SMF treatment and their interrelationship with the structural-functional state of the membrane were reported. The presented work would have future fundamental applications in biomedicine.

## 1 Introduction

Knowledge of the mechanisms of action and biological effects of static and variable magnetic fields has significantly increased (Fei et al., 2023; Ji and Zhang, 2023; Makinistian et al., 2023). The SMF action on living cells depends on parameters of the magnetic field, such as homogeneity, intensity, and exposure time (Ji and Zhang, 2023). The number of applications of magnetic fields in research, industry, and medicine has increased (Lei et al., 2020; Wu et al., 2022; Zhang et al., 2022). The development of new technologies for energy production and storage is essential for studying the biophysical aspects of magnetic fields.

The effects of static magnetic field (SMF) (≥0.1 T) are studied on biological membranes because of their large biological application in practice, especially in the increase of reactive oxygen species (ROS) levels (Calabrò et al., 2013; Vergallo et al., 2014; Zablotskii et al., 2014; Wang and Zhang, 2017; Mustafa et al., 2019; Zadeh-Haghighi and Simon, 2022). Magnetic fields have been used in diagnostics such as magnetic resonance imaging (Mustafa et al., 2019). The physical interaction between SMFs and biosystems is due to the following mechanisms: 1) electrodynamic interactions with ionic solutions; 2) magnetomechanical effects and the orientation of magnetically anisotropic structures in constant fields and the behavior of paramagnetic and ferromagnetic materials in gradient magnetic fields; and 3) effects on negatively and positively charged molecules, which are due to unbounded electrons in the outermost shells of the blood (Bansi et al., 2018). Intact erythrocytes are oriented with their disk planes parallel to the magnetic field because of diamagnetism (Chaudhury, 2021) of cell membrane components, such as transmembrane proteins (band 3, glycophorin) and the lipid bilayer (Higashi et al., 1993; Higashi et al., 1995). Paramagnetic interactions (Chaudhury, 2021) between the blood flow and magnetic fields have been reported (Shiga et al., 1993). Red blood cells can be paramagnetic or diamagnetic depending on their oxygenated state (Bansi et al., 2018).

Erythrocytes are used as models to study the properties of different biological membranes because they possess only plasma membranes and no cellular organelles. They easily produce “ghosts” by releasing erythrocytes from hemoglobin (Hoffman, 1992).

The electrophoretic mobility of the cells gives information about the dynamics of the surface electrical charge on the outer membrane surface, i.e., the average amount of the electrical charge that is generated on the outer surface of the cell membrane of the erythrocytes is calculated. The surface electric charge is determined from the zeta potential of the cell by making the approximation that the electrokinetic potential is approximately equal to the surface or electrostatic potential of the cell according to Gouy-Chapman theory. It explicitly states that the value of zeta potential depends on the ionic strength. Therefore, erythrocytes were placed in a medium with isotonic ionic strength to follow the change in the electrokinetic parameters in the erythrocyte membrane upon SMF exposure. This theory is used in the calculation of the surface charge. The electric charge distributed over the surface of biological membranes is essential in the regulation of molecular membrane processes and in the interaction of biological cells with external influences, such as magnetic fields.

The electrokinetic (zeta) potential, an electrochemical property of erythrocyte surfaces, is determined by the net electrical charge of surface-exposed molecules. The surfaces of erythrocyte membranes carry a net negative charge at a neutral pH, and sialic acid residues are responsible for most of the negative charges on the cell surface (Mehrishi and Bauer, 2002; Karemore and Avari, 2019; Doltchinkova et al., 2021). The erythrocyte membrane is surrounded by a fixed layer of cations and coions in the medium (Galassi and Wilke, 2021) by a diffuse double layer (DDL) of a mixture of cations and anions. DDL describes the variation of electric potential near a charged surface, and behaves as a capacitor. Within the diffused layer, the Brownian motion of cells and the flow of medium create a shear plane, which separates unfixed ions from those ions closely associated with the fixed layer. The potential at the shear plane is defined as the zeta potential (Tokumasu et al., 2012). Several pathologies are linked to structural and mechanical changes within the cytoplasmic membrane and the cell’s mechanical properties in dependence on red blood cells lipidome (Himbert and Rheinstädter, 2022). Normal young red blood cells bear 43–55 × 10^6^ molecules of sialoglycoprotein sialic acid and 10^7^ electron charges, according to the Gouy-Chapman screening theory (Mehrishi, 1972). β-thalassemia erythrocytes are characterized by a decrease in glycoproteins and sialic acids, which predetermines the reduced deformability of the cells (Huang et al., 2011; Mehrishi and Risso, 2016). According to Yuan et al., 1995; Cao and Galanello, 2010, there is an accumulation of unmatched α or β-globin chains in thalassemic red blood cells that lead to hemolysis and varying forms of anemia. Inheriting one β-thalassemia gene (heterozygote) frequently leads to no or mild hypochromic microcytic anemia or an elevated or normal red blood cell count (Mehrishi and Risso, 2016). Heterozygous β-thalassemia has been suggested to increase the risk of autoimmune diseases (Galanello and Origa, 2010; Altinoz et al., 2012). Heterozygous thalassemia protects against multiple sclerosis patients (Cikrikcioglu et al., 2016). It has been observed that β-thalassemia erythrocytes possess lower electrophoretic mobility in physiological media at pH 7.4, owing to the diminished amount of sialic acid on the thalassemic erythrocytes and reduced surface electrical charge (Mehrishi and Risso, 2016; Hristova-Avakumova and Doltchinkova, 2018). The negative charge on the erythrocyte surfaces is believed to prevent cell aggregation. The net surface electrical charge varies in different diseases and is genetically determined by electrokinetic (zeta, ζ) potential values. The distance from the closest approach governs the physicochemical interactions of sialic acid with monocytes and macrophages during erythrophagocytosis (Mehrishi and Risso, 2016). The outer surface of erythrocyte membranes possesses net negative charges with an approximate value of σ = −0.0181 Cm^−2^ (Doltchinkova et al., 2021). SMF exposure of erythrocytes in normal and β-thalassemia conditions leads to the aggregation of cell membranes, which is unique for diagnosing erythropaties (Dybas et al., 2022).

Since highly conductive systems efficiently interact with magnetic fields, it is of significant interest to study the possible mechanisms of the interaction of magnetic fields with biological cells by the example of such a model system as a charged erythrocyte membrane (biconcave disks: (6.2–8.2) × (2–2.5) × (1–1.8) µm in the middle (Gallagher, 2005; Himbert and Rheinstädter, 2022). The magnetic fields can interact directly with moving electric charges (ions, electrically non-compensated proteins, etc.) by the well-known Lorentz force deflection and magnetic materials with non-compensated spins in specialized magnetoreception systems (complexes containing Fe, for instance) in membranes (Roumenin, 1994). Hence, the magnetic field influences the kinetics of reactions with radical-pair intermediates (McLauchlan and Steiner, 1991; Grissom, 1995). SMF influence on the erythrocytes in norm and β-thalassemia is defined as a spontaneous chemical reaction as a result of SMF treatment of the biological system. The upper reaction is expected to occur spontaneously where no additional energy (in the form of heat) input is required in order for the reaction to occur, and the sum of all chemical interactions must result in a negative change in electrostatic free energy (∆G_electrostatic_) (Du et al., 2016; ImmunoChemsitry Technologies, 2023). There are a number of methods for the analysis of thermodynamic parameters of biological systems, through which molecular modeling of *in vitro* processes is possible with the aim of reactivating enzyme systems inhibited by various nerve agents (Giacoppo et al., 2015; Du et al., 2016). The calculation of the electrostatic free energy thermodynamic parameter following the application of a static magnetic field of different intensities can be useful in the selection of new reactivators of biological systems to normal states. The present study focused on the direct contribution of an SMF (2.0 T) to erythrocyte membrane epifluorescence after FITC-Concanavalin A or FITC-Wheat Germ Agglutinin labeling to study receptor binding after SMF treatment. Low-frequency periodic electromagnetic exposure induces a reduction in the ligand-receptor lifetime of the lectin, irrespective of the field waveform. A decrease in the mitogenic capability of the ligands has been observed (Chiabrera et al., 1984). The investigation of the contribution of SMFs to lipid peroxidation processes is another point of view toward understanding the influence of SMF at different magnetic intensities on erythrocytes from healthy subjects and patients with heterozygous β-thalassemia.

Because of their precisely defined structure, human erythrocyte membranes from healthy subjects and β-thalassemia patients are used in the study as model systems to examine the SMF effect on the cellular membrane in dependence of SMF intensity of exposure and iron content in the blood. Increased knowledge of the influence of SMF on biophysical properties has been reported to have significant therapeutic potential (Elblbesy, 2017; Zhang et al., 2017; Marycz et al., 2018; Zhang and Tian, 2020).

The effect of SMF on the physicochemical properties of erythrocytes under normal and pathological conditions was also investigated. In this article, we report i) the potential of using SMF treatment on electrokinetic, light scattering, and lipid peroxidation parameters of erythrocytes in norm and β-thalassemia and ii) clarifying the SMF (2.0 T) alteration in the morphology of erythrocyte membranes from healthy subjects and β-thalassemia patients by fluorescence microscopy.

## 2 Materials and methods

### 2.1 Materials

All chemicals were of analytical grade. Na_2_HPO_4_, disodium phosphate, NaH_2_PO_4_, sodium phosphate monobasic monohydrate, KH_2_PO_4_, monopotassium phosphate, FITC CoA, Concanavalin A-Fluorescein Isothiocyanate from *Canavalia ensiformis* (Jack Bean) type IV, FITC WGA, Wheat Germ Agglutinin- Fluorescein Isothiocyanate from *Triticum vulgare* and NaCl were purchased from Sigma-Aldrich (St Louis, MO, United States). DAPI, 4´, 6-Diamidino-2´-phenylindole dihydrochloride (10236276001) was purchased from Roche Diagnostics GmbH, Mannheim, Germany. Phenylmethanesulfonyl fluoride (PMSF) was purchased from Fluka Chemie, AG. KCl was purchased from Riedel-de-Haën AG, D-3016 Seelze 1. Glycerol (1, 2, 3-Propanetriol, Glycerin) was obtained from NBCo Biochemicals (Division of ICN Biochemicals, Inc., Cleveland, Ohio 44128).

### 2.2 Erythrocyte preparations

Venom blood samples are taken from healthy human subjects and β-thalassemia patients using an EDTA-anticoagulant vacutainer study. The present study complied with the ethical regulations and legislation in Europe and Bulgaria. The experiment was performed in compliance with the WMA Declaration of Helsinki and the Ethical Principles for Medical Research Involving Human Subjects (https://www.wma.net>wma-declaration-of-helsinki-ethical-principles-for-medical-research-involving-human-subjects/09/06/2022). Written informed consent was obtained from all subjects recruited for this study. Ethics Committee of Sofia University, “St. Kliment Ohridski”, Approval Codes: RD-04-91, 25 February 2022, and RD-04-68, 2 February 2023.

Blood samples were collected from the following groups according to the principles of the World Medical Declaration of Helsinki (https:// www.wma.net/>wma-declaration-of-helsinki-ethical-principles-for-medical-research-involving-human-subjects/09/06/2022):

1. Nine healthy subjects with normal hematological values and indices;
2. Nine heterozygous β-thalassemia patients diagnosed by hemoglobin electrophoresis studies with HbA_2_ levels higher than 4.95%, up to (5.78%–6.02%).

Blood samples were centrifuged (1,000 × g, 5 min, 4°C). Plasma and buffy coat were discarded, and the erythrocytes were washed three times under the same conditions of centrifugation with two volumes of phosphate buffered saline (PBS), pH 7.4 (PBS: 10.1 mM Na_2_HPO_4_, 1.8 mM KH_2_PO_4_, 136.9 mM NaCl, 0.2 mM KCl) at 4°C. After centrifugation of the blood samples, the erythrocyte ghosts were prepared using the method described by Burton et al. (Burton et al., 1981; Yoshimine et al., 1992) with modifications. The erythrocyte ghosts were performed by hemolysis and washing the erythrocytes (500 µL) in 15 mL of 5 mM sodium phosphate buffer (Na_2_HPO_4_/NaH_2_PO_4_, pH 8.0) at 4°C for 20 min. This is followed by the centrifugation in the same solution at 17500 × g (Janetzki 24, Engelsdorf (Bez. Leipzig), Germany) for 30 min. The pellet was washed with 17500 × g (Janetzki Engelsdorf (Bez. Leipzig), Germany) in 15 mL 2.5 mM sodium phosphate buffer, pH 7.75 for 30 min at 4°C. The pellet was washed twice in 15 mL of 1.25 mM sodium phosphate buffer, pH 7.6 for 30 min at 4°C. The final pellet of erythrocyte membranes (erythrocyte ghosts) was suspended in 100 µL of cold Sorensen’s phosphate buffer (1/15 M Na_2_HPO_4_/NaH_2_PO_4_, pH 7.4), 0.7 mM PMSF at 4°C.

The erythrocyte membranes were stored at 4°C for no more than 4 h before sample preparation for microelectrophoretic measurements. Erythrocyte ghosts were stored at −20°C in the presence of 20% glycerol before sample preparation for the fluorescence microscopy experiments. The protein concentration was measured as previously described (Lowry et al., 1951).

### 2.3 Microelectrophoretic measurements

Electrophoretic mobility (EPM) measurements were performed using particle electrophoresis (microscopic (visual) microelectrophoresis) with an OPTON Cytopherometer (Feintechnik Ges., Germany). Electrophoretic migration is measured in a rectangular chamber and platinum electrodes at a constant electric field of 5 mA and 25°C. The movement of the cells over a known distance (16 μm) was timed for forward and backward (reversed field) runs. The erythrocytes were observed under a light microscope connected to a Sony video camera at a magnification of 2000 ×. The cells were observed on a JVC monitor (Victor Company of Japan, Ltd., Yokohama, Japan). Erythrocytes of approximately equal size were measured. The results are expressed using the EPM ( u per 10^–8^ m^2^ V^−1^s^−1^ ± standard deviation for each sample). The standard deviation of the electrophoretic mobility *u* was 2%–5%. The electrical conductance and viscosity of different media, including erythrocytes, were measured using a CyberScan PC 510 (Eutech Instruments, Singapore) pH/conductivity meter and a Rheo (Germany) viscometer, respectively. Values represent the mean of three independent measurements (54–96 erythrocytes) with three replicates each. The zeta potential (ζ) is calculated from the electrophoretic mobility, *u*, using the Helmholtz-Smoluchowski equation (Delgado et al., 2007):

$$\zeta =\frac{\eta u}{\varepsilon_{r}\varepsilon_{0}},$$ (1)

where $\zeta \;\left(electrokinetic,zeta\;potentail\right)$ is in units of mV; $\varepsilon_{r}=78.5\;\left(at\;25℃\right)$ is the relative dielectric permittivity of the aqueous phase; $\varepsilon_{0}=8.8542\times 10^{-12}\;Fm^{-1}$ is the permittivity of free space, and η=0.001393 Pa.s is the viscosity of the phosphate buffered saline (PBS), pH 7.4 at 25°C as in McLaughlin, (1977).

The electrostatic potential in the aqueous phase of the membrane surface at x = 0 and surface charge density ( $\left.\sigma \right)$ is given by:

$$\frac{136.6\;x\;\sigma }{\surd C}=\sin h\left(\frac{z\Psi_{o}}{51.38}\right),$$ (2)

where $\Psi_{o}$ is in mV (McLaughlin, 1977). The surface electrical charge is expressed as Cm^−2^ (in Coulombs per meter squared; The Coulomb is defined as the quantity of electricity transported in one second by a current of one ampere). The value of surface charge density is calculated using the assumption that $\zeta ≅\Psi_{o}$ (McLaughlin, 1977).

### 2.4 Electrostatic free energy

The electrostatic Coulombic part of the electrical work done in charging up the surface of the erythrocyte membrane can be calculated. The location of charged groups plays a role in determining the types of interactions in which they participate, and the contributions they make to the electrostatic free energy of the SMF-treated erythrocyte cells in norm and pathology (McLaughlin, 1977; Cevc, 1990).

In the linear regime of Gouy-Chapman approximation the dominant contribution to the electrostatic free energy comes from the internal energy, which is proportional to the volume of the integral of the square of the electric field (Cevc, 1990):

$$G_{elec}^{s}=G_{Gouy}=-\int_{0}^{\Psi }\sigma d\Psi_{0}$$ (3)

The integral in Eq. 3 is due to the electrical work done in moving ions from the bulk to a surface with a final net surface charge density $\sigma_{el}$ , electrostatic surface potential $\Psi_{0}$ , where electrostatic potential Ψ ( σ , $\sigma_{p}=0$ ), and free energy $G_{elec}$ .

The Coulombic free energy of most membranes is only small, below 2 kJmol^−1^, owing to the interfacial structure effects (Cevc, 1990). The electrostatic free energy per 1 cm^2^ of the double electrical layer near the outer surface of the erythrocyte membrane was calculated:

$$G_{Gouy}=–\;\frac{8N_{A}kT}{\lambda }\left[\cosh\left(\frac{y_{0}}{2}\right)\;–\;1\right],$$ (4)

where $\sigma ={\left(\frac{2N_{A}DkT}{\pi }\right)}^{½}\sinh\left(\frac{y_{0}}{2}\right)$ , $y_{0}=\frac{Ze\Psi_{0}}{kT},e=1.6021\times 10^{-19}C$ is the elementary electronic charge, Z is the ion valence, $k=1.3805\times 10^{-2}JK^{-1}$ is the Boltzmann constant, $N_{A}=6.022\times 10^{23}{mol}^{-1},$ is the Avogadro constant, T=298. 15°K is the absolute temperature.

The Debye-Hückel length by the general definition in all electrostatic theory:

$$\lambda ={\left(\frac{{2e^{2}N}_{A}n_{0}Z_{i}^{2}}{\varepsilon kT}\right)}^{\frac{1}{2}}={\left(\frac{{2e^{2}N}_{A}cZ_{i}^{2}}{1000\varepsilon kT}\right)}^{\frac{1}{2}}≅0.328\cdot 10^{-10}\sqrt{cZ^{2}}\;\left[m^{-1}\right],$$ (5)

where the summation goes over all ionic species and the factor $10^{3}$ appears because the bulk ion concentration is normally given in moles per liter; $n_{0}i$ = concentration of the n_oi_(x) species of the i^th^ - ion at definite point x with potential E(x) at 25°C; $Z_{i}$ = electrolyte valence of the i^th^ ion; c = electrolyte ionic strength = bulk concentration of the i^th^ species, ideally for x →∞, but practically just far enough away from the surface charge region.

### 2.5 Static magnetic field treatments

#### 2.5.1 Permanent magnetic field system for magnetic measurements of samples with β-thalassemia

**FIGURE 1:**
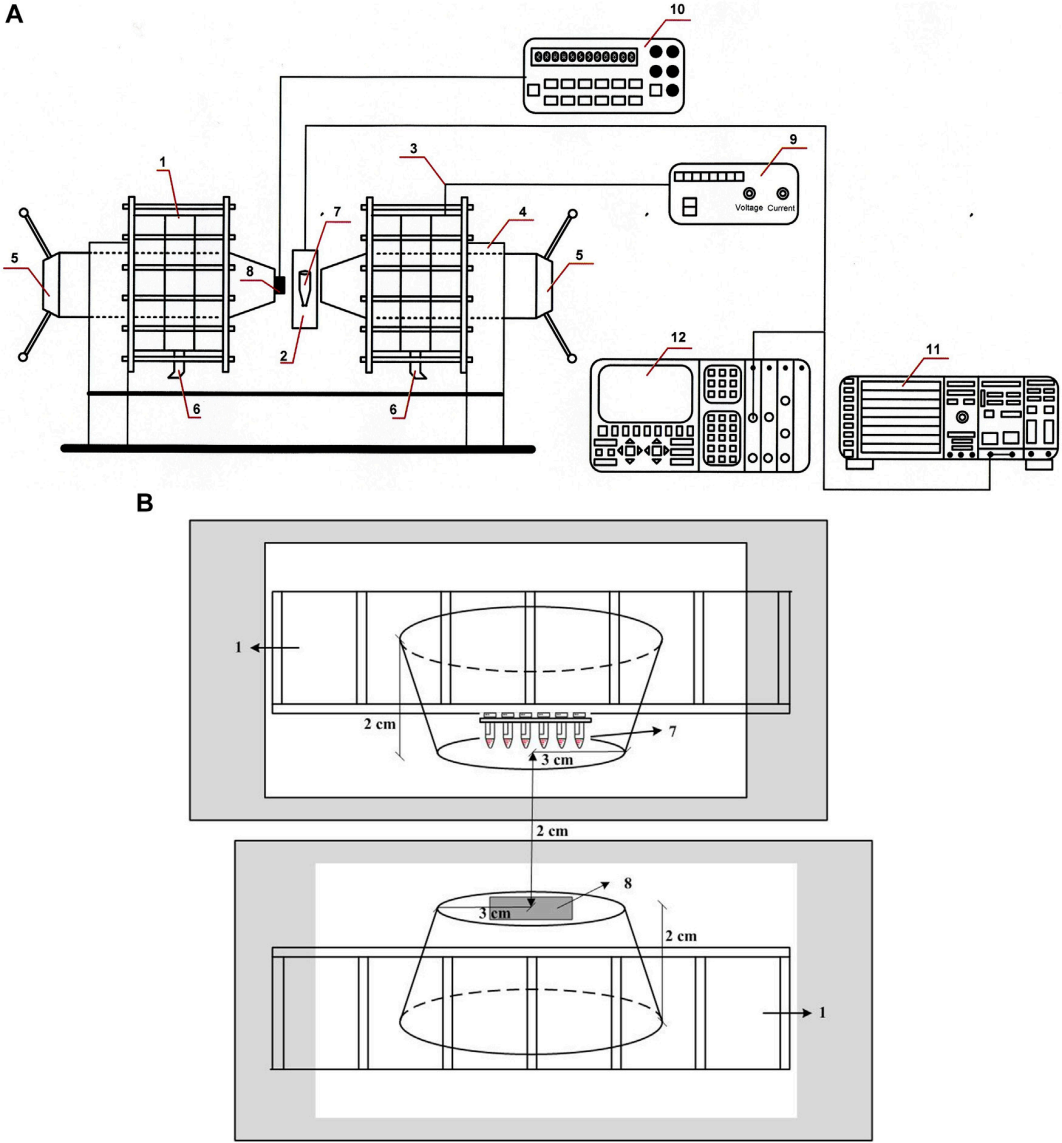
**(A)** Experimental setup used for the SMF exposure of erythrocyte samples. 1-Weiss U type electromagnet with water cooling; 2-Eppendorf tube (1.5 mL) containing the sample; 3-Constant current generator; 4-Electromagnitic yoke; 5-Perpendur Electromagnetic poles (an iron mild magnetic alloy containing 48%–50% of Co with magnetic induction maximum of 2.4 T), ɸ = 60 mm; 6-Water cooling; 7-Samples; 8-Hall sensor type KSY 14 calibrated for determining the direction and induction field B; 9-Supply voltage block HP 34401A; 10-Digital measure block HP 34401A; 11-HP 1980B oscilloscope; 12-Philips spectral analyzer (model #PM3360). **(B)** Experimental scheme used for SMF treatment of erythrocyte samples. The erythrocyte sample tubes are placed on a Plexiglas stand with six wells in which the Eppendorf tubes are placed in a vertical position, attached to one pole of the magnet in a thermostatically adjustable chamber. 1-Weiss U type electromagnet with water cooling; 7-Samples; 8-Hall sensor type KSY 14 calibrated for determining the direction and induction field B. Radius of distance between poles (inter-pole distance) is 2.0 cm; radius of working area of truncated cone is 3.0 cm; height of truncated cone is 2.0 cm.

The treatment of the erythrocyte suspension after washing three times in the suspending buffer is conducted in a volume of 250 µL (Hematocrit 15%). The treatment of the blood samples with the SMF intensities (0.1–2.0 T) is conducted with a specially made Plexiglas stand with six wells in which the Eppendorf tubes are placed in a vertical position, attached to one pole of the magnet, and the exposure is applied, or samples with erythrocytes are conducted and the corresponding magnetic field without being treated with it (sham-controlled samples). Exposure time is 15 min for sham-expressed and exposed in SMF samples at a constant temperature of 25°C. The distance between the Eppendorf tubes with the appropriate erythrocyte samples and the support was adjusted to guarantee the magnetic field strength in the erythrocyte tubes. Thus, for each exposure, the erythrocytes were treated homogeneously during the same period. The magnetic field traversed a layer or several layers of cells in the same way, that is, erythrocyte grouping or density did not change the field. The magnetic field was adjusted using a customized screw, and the magnetic field was measured using a Hall effect Gaussian meter (Figures 1A, B).

Magnetic experiments with samples containing normal erythrocytes or β-thalassemia were conducted in permanent magnetic fields. Regardless, the most commonly used range of magnetic inductions for practical purposes is up to −1.0 T ≤ B ≤ 1.0 T, in which the technological task of generating the field B is significantly eased. However, studying the mechanisms and processes of the effect of field B on the blood substrate requires an induction range of 1.5–3.0 T. At these high values of field B, the factors influencing the exchange interactions of hemoglobin clusters with magnetic factors are detailed. In the presented experiments, a water-cooled Weiss-type electromagnet was used, located in the Laboratory of “Magnetic Measurements” of the Institute of Robotics-Bulgarian Academy of Sciences (IR-BAS) (Figure 1A). A cast magnet from magnetic alloys of the Iron-Nickel-Aluminum system with other additions containing cobalt from the Ferromagnet Factory -Sofia was used. The manufactured magnet is from pure original materials. The metal is cast into molds from sand mixtures, the composition of which is controlled by the Ferromagnet Factory–Sofia. The cast form is subjected to heat treatment, after which certain magnetic properties are obtained (permanent magnets). The erythrocyte samples are shown in Figure 1B, where they are placed on a Plexiglas stand with the bottom of the eppendorf tubes sinking 3.7 cm down from the stand. The thermostatically adjustable chamber is also presented, with the upper part of the eppendorf tubes located next to each other and placed in the central part of one concentrator in the shape of an inverted cone opposite the other concentrator where the Hall sensor is placed. The samples were placed in a thermostatic chamber to maintain the same temperature of impact with the static magnetic field and the temperature of measuring their electrophoretic mobility. The height of the camera is 4 cm, its length is 5.8 cm, and the width of the plexiglass stand is 9 mm. The Gauss meter was placed at the center position of the camera to measure the magnetic flux density. An advantage of this system is the high coercive force of the used magnetically soft material. With an electric power of only 1.2 kW, the generated field B at an inter-pole distance of 20 mm is 2.2 T, and at 15 mm, the maximum induction is 3.5 T. The laboratory-made equipment minimized the possible heating of the electromagnet housing from the inevitably penetrating 50 Hz component and eliminated the characteristic hum, including the uncontrolled heating of thalassemia samples, which is a problem in transformation-type systems with high-power supplies. The body (yoke) of the magnet was composed of densely packed lamellae with a thickness of 1 mm and covered with a suitable varnish. The total weight of the electromagnet is approximately 450 kg. The coils for generating the magnetic field contained 2,500 turns of copper wires. They were divided symmetrically on both sides of the concentrators with 6 mm thick copper cylindrical segments cooling them with running water located between the coils. A permanent control of the electromagnet calibration was performed using a reference magnetometer type Metrolab-THM 7025, Schaefer, Switzerland). The information about the direction and value of magnetic field B was obtained from a high-precision n-GaAs miniature Hall sensor (type KSY 14, Siemens Company), specially mounted on one of the electromagnet poles. The working diameter of the two truncated cone concentrators in field B was 60 mm, and the bevel angle of the concentrators was 50°. Therefore, at the center of the distance between the poles for structures with a volume of 7–10 mm^3^ in the experiments, a high degree of homogeneity of magnetic field B is maintained. The electromagnet was powered using a stabilized current-source-type HP 6010A DC (Hewlett-Packard Corp.). The error in the measurement of the magnetic induction is the sum of the individual errors of the sensor registering field B, the signal processing electronics, and the error in the calibration of the measuring element of Hall KSY 14. Practically, each of these error is approximately ±0.3%. It follows that the total error in the determination of the magnetic induction B was no greater than ±1.0%. This is the first study of a permanent magnetic field B conducted on samples with β-thalassemia erythrocytes, where the exposure time of 15 min was used. The conductivity of the erythrocyte suspension medium after treatment with an SMF did not change compared to that of untreated cells. Thus, conductivity is not the factor that affects the electrokinetic properties of erythrocytes in norm and beta-thalassemia. It should be noted that erythrocytes without and after exposure to an SMF were diluted 500 times in the suspension medium before their electrophoretic mobility was determined. In this way, the optimal density of the cells and precise determination of their electrokinetic properties under the influence of the SMF of different intensities are measured.

Preliminary studies were conducted on the influence of different exposure times and SMF intensities (1, 5, 10, 20, 30, and 60 min) on the biophysical parameters of healthy erythrocytes and β-thalassemia patients. Treatment of the biological samples for 15 min was chosen so that there would not be a significant difference in the time after treatment in the field. SMF treatment was selected as 15 min, which is the optimal treatment to avoid aging the sample in the subsequent electrokinetic measurements, with the blood being used up to 4 h after centrifugation in the isotonic PBS solution. The experiments are conducted at a temperature of 25°C in a homogenous magnetic field. A magnetic field was applied perpendicular to the tubes containing the erythrocyte suspension (microcentrifuge polypropylene tubes, Sigma-Aldrich). The tubes without magnetic field action are maintained at 25°C. At the end of exposure, the final erythrocyte suspensions (exposed and unexposed to SMF) are stored in ice at 0°C before measurements.

### 2.6 Fluorescence microscopy studies

*In vitro* fluorescence microscopy was performed using a BX50-FLA (Olympus, Tokyo, Japan). Fluorescein isothiocyanate (FITC)-labeled lectin (CoA) and wheat germ agglutinin (WGA) were used. Concanavalin A FITC labeled from *C. ensiformis* (Jack bean) Type IV, FITC content 3.6 mol/mol lectin (Mol.Wt of lectin approx. 102,000) has an affinity for terminal α−D−mannosyl and α−D−glycosyl residues. CoA-FITC inhibitory carbohydrates are α−Methylmannoside and α−Methylglucoside. Lectin from *Triticum vulgaris* (Wheat Germ) fluorescein isothiocyanate labeled contains 2 mg protein containing 2.2 mol FITC per mole lectin (Mol. Wt of lectin approx. 36,000). The secondary structure of the protein (FITC-WGA) is rich in strands and contains carbohydrate-binding sites on its surface. It is used for the fluorescence detection of glycoproteins containing β (1 β 4)−N-acetyl-D-glucosamine. WGA is not blood-specific but has an affinity for N−acetyl−β−D−glucosamine oligomers. WGA does not contain protein-bound carbohydrates. FITC-Con A (MW 102,000 Da) or FITC-WGA (MW 36,000 Da) labeling was dissolved in distilled water at 2 mg/mL as a stock solution in dark tubes. The solution was diluted with PBS (pH 7.4–20 µg/mL) before use. Erythrocyte membranes were then transferred to the staining solution. Erythrocyte ghosts were exposed to SMF (2.0 T) for 15 min at 25°C. After 60 min incubation with the staining solution, erythrocyte ghosts (2 × 10^6^ cells/mL) were washed three times with PBS (15 min, 12,000 × g) and examined under an epifluorescence microscope (BX50-FLA, Olympus, Tokyo). Nuclear counterstain DAPI (4´,6-Diamidino-2´-phenylindole dihydrochloride; 5 mg/mL stock solution; Excitation at 358 nm/Emission at 461 nm) was used to assess gross cell morphology (Klinge et al., 2022). DAPI staining allowed multiple uses of cells eliminating the need for duplicate samples (Tarnowski et al., 1991).

### 2.7 Light scattering measurements

The intensity of light scattering (LS) at 90° and λ=480 nm from the erythrocyte suspension is registered using a laboratory-made apparatus using Specol 10 Spectrophotometer (Carl Zeiss, Germany) and titration attachment type Ti. Cut-off filters (red glass filter with λ≥640 nm and blue-green glass filter with λ≤600 nm) are used. The reaction medium used in the LS experiment contained PBS (pH 7.4). The scattering level at an angle of 90^o^ represents the level of cell aggregation.

### 2.8 Detection of malondialdehyde (MDA)

Malondialdehyde (MDA) content was measured according to (Halliwell and Gutteridge, 1999) with slight modifications. The erythrocyte suspensions (500 μL erythrocytes in PBS, pH 7.4, 2 mM NaN_3_, Ht = 20%) are homogenized in 400 μL of 28% trichloroacetic acid (TCA) and centrifuged at 12,500 × g for 15 s. After centrifugation, 1 mL of the supernatant was mixed with 500 μL of 1% thiobarbituric acid (TBA) in 20% NaOH, and the mixture was incubated in boiling water for 15 min. The reaction was terminated by cooling the samples in an ice bath. In the TBA test, MDA reacts to generate a colored product. In the acidic solution, the product absorbed light at λ = 532 nm. The MDA content was estimated using an extinction coefficient of 154 mmol L^−1^ cm^−1^. Lipid peroxidation in erythrocyte membranes is determined by the production of thiobarbituric acid reactive substances (TBARS) and expressed in mol L^-1^ (Halliwell and Gutteridge, 1999).

### 2.9 Statistical analysis

Electrophoretic mobility, zeta potential, and surface electrical charge data are expressed in ***u*** x 10^–8^, m^2^V^−1^s^−1^, ζ, mV, and σ, Cm^−2^, respectively. The light scattering levels were expressed as arbitrary units. All electrokinetic and electrostatic free energy data are reported as the mean ± SD. The results were averaged from three independent experiments, each with three replicates. Statistical differences between means were determined using Analysis of Variance (ANOVA). One-way analysis of variance was performed using the Holm-Sidak method to compare the significance of the treatments taking *p* ≤ 0.05 as significant, *p* ≤ 0.01 as highly significant, and *p* ≤ 0.001 as extremely significant. Statistical analyses were performed using Minitab.v.17.

## 3 Results

### 3.1 Effects of SMF on the electrokinetic parameters of erythrocytes

**FIGURE 2:**
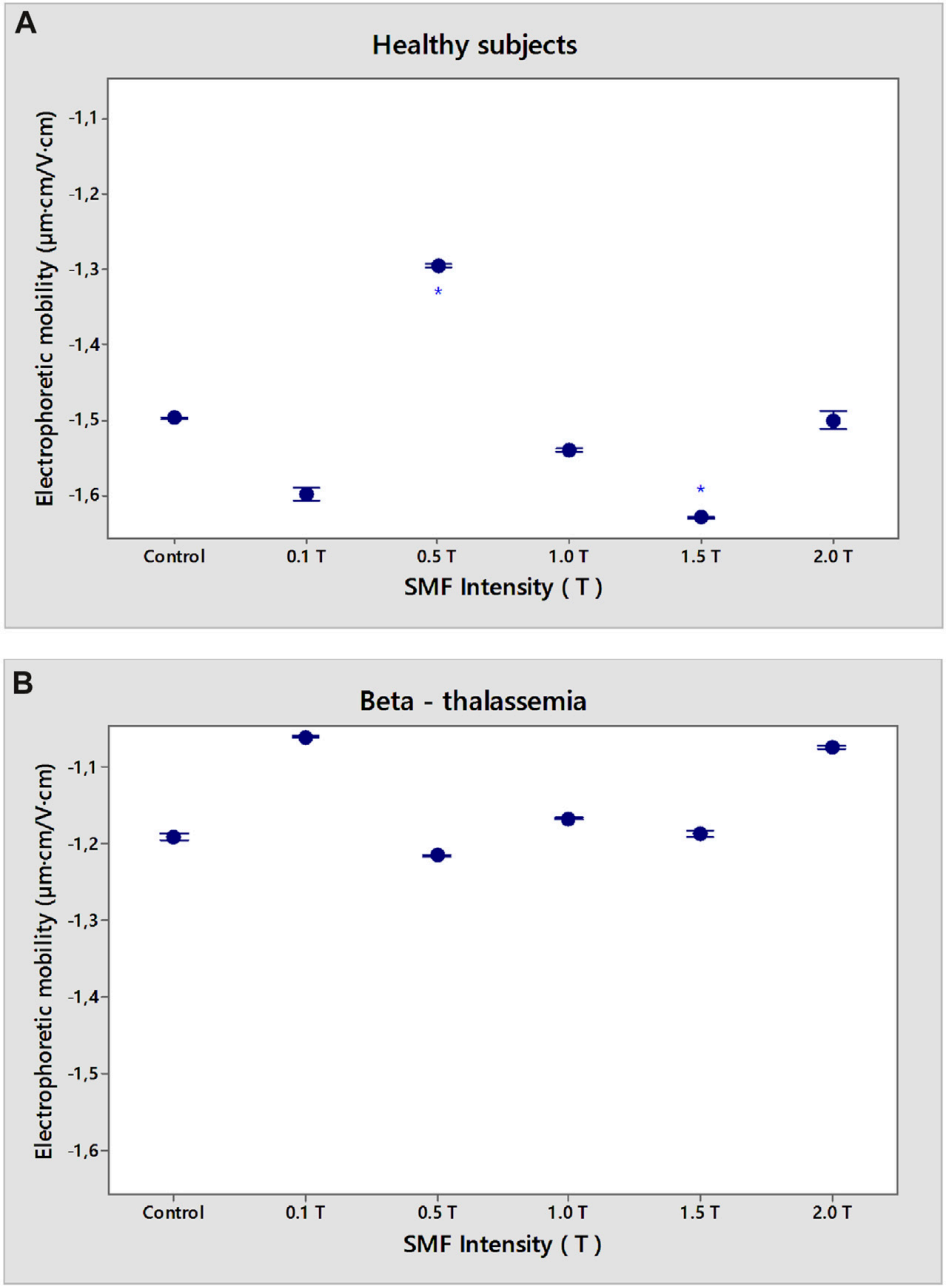
Dependence of SMF (0.1–2.0 T) on electrophoretic mobility (EPM) of erythrocyte membranes in norm **(A)** and β-thalassemia **(B)**. The solution contains phosphate buffered saline (PBS), pH 7.4. EPM of erythrocytes in norm and pathology is measured after 15 min-SMF pre-exposure at 25°C. Values are expressed as mean ± SD (*n* = 54–75) of three independent measurements, each of three replications *p < 0.05.

**FIGURE 3:**
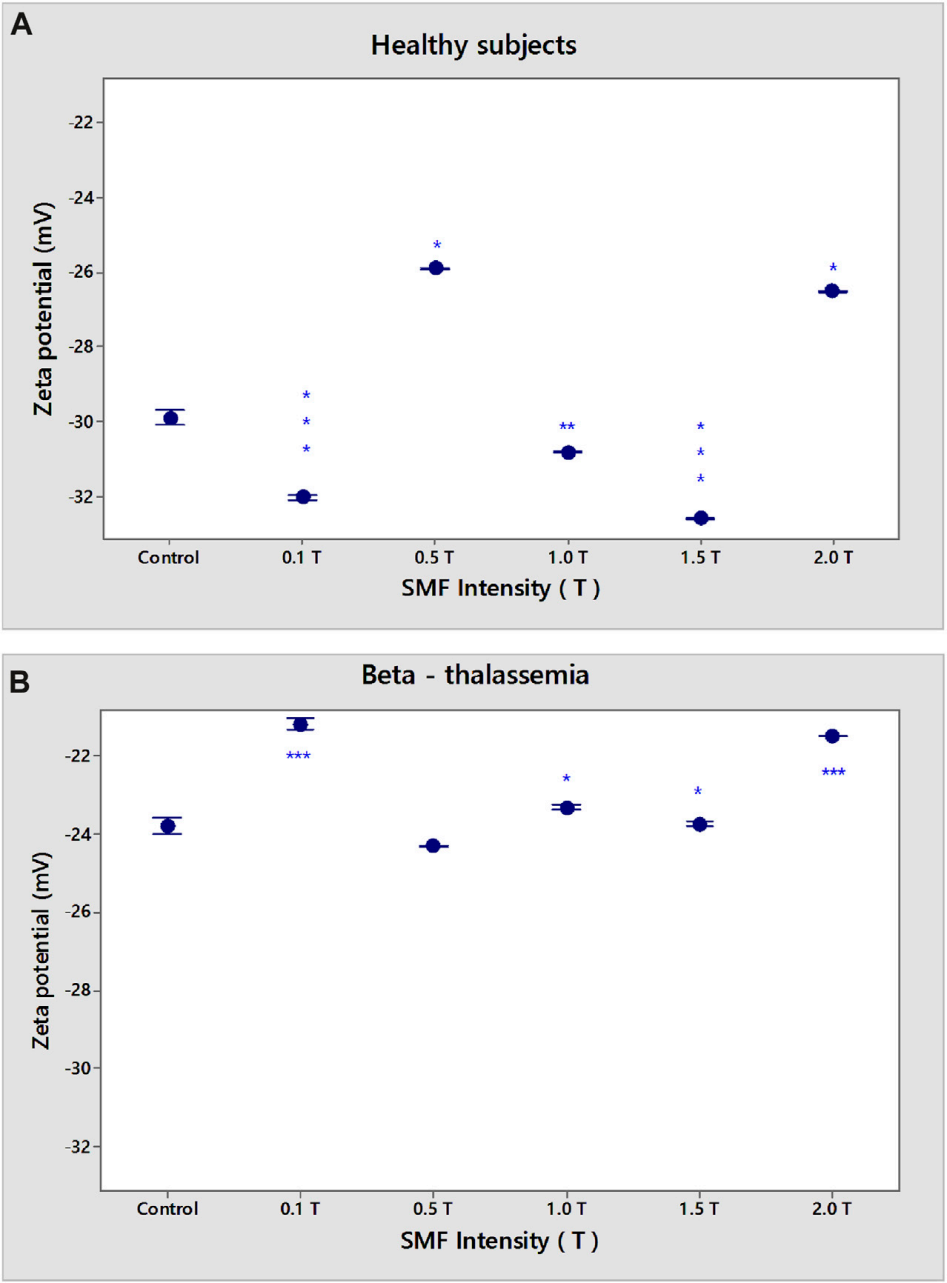
Dependence of SMF (0.1–2.0 T) on zeta potential (ζ) of erythrocyte membranes in norm **(A)** and β-thalassemia **(B)**. The solution contains phosphate buffered saline (PBS), pH 7.4. EPM of erythrocytes in norm and pathology is measured after 15 min-SMF pre-exposure at 25°C. Values are expressed as mean ± SD of three independent measurements, each of three replications. *p < 0.05; **p < 0.01; ***p < 0.001.

**FIGURE 4:**
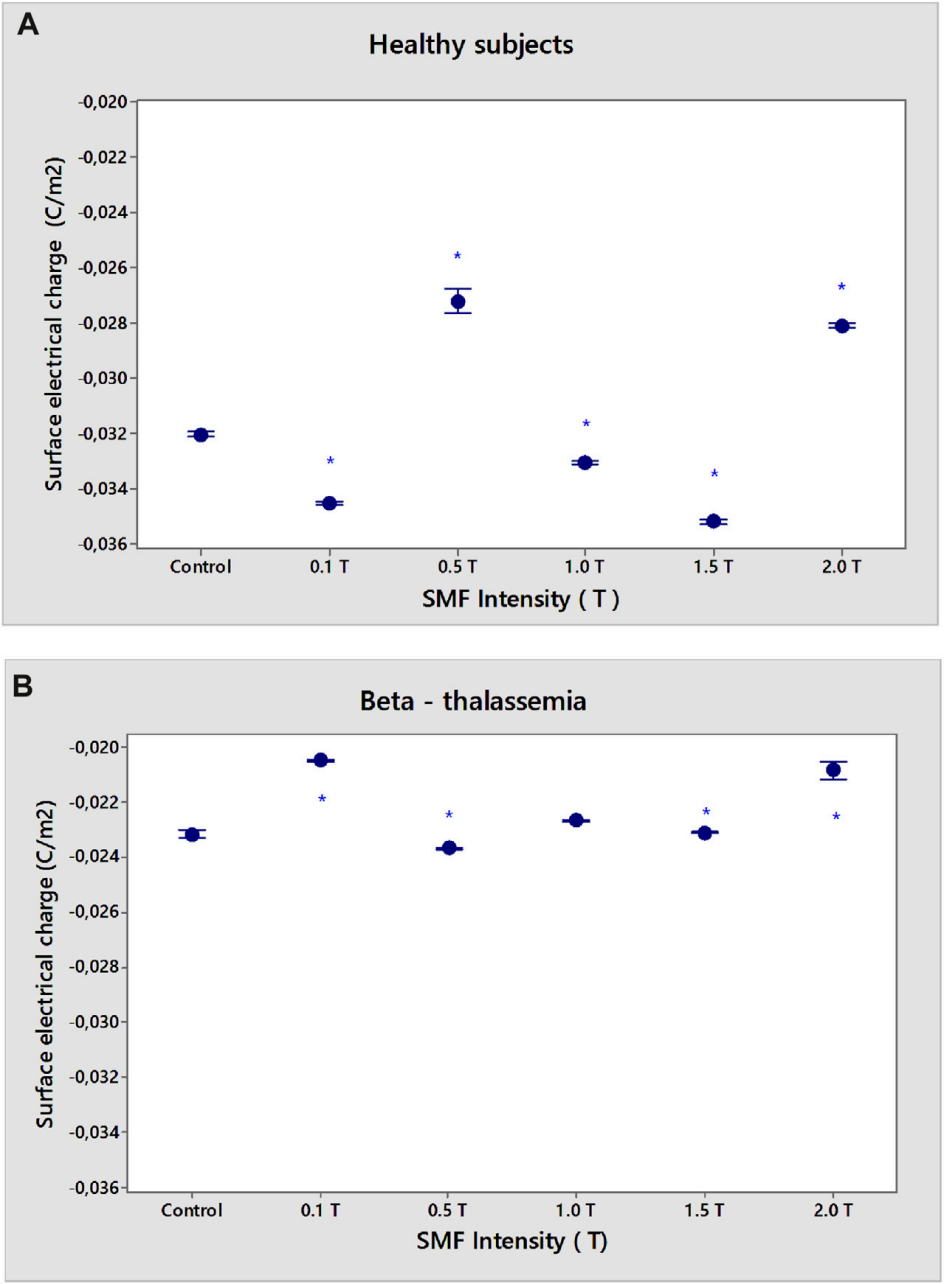
Dependence of SMF (0.1–2.0 T) on the surface electrical charge (σ) of the erythrocyte membranes in norm **(A)** and β-thalassemia **(B)**. The solution contains phosphate buffered saline (PBS), pH 7.4. The EPM of the erythrocytes in norm and pathology is measured after 15 min-SMF pre-exposure at 25°C. Values are expressed as mean ± SD of three independent measurements, each of three replications. *p < 0.05.

Erythrocytes from healthy subjects and β-thalassemia patients were used as model systems to evaluate the effect of SMF on membrane surface charge. The SMF treatment was applied for 15 min. The EPM of erythrocytes after exposure of norm and β-thalassemia samples to SMF (0.1–2.0 T) is shown in (Figures 2A, B). There was a decrease in the EPM values of healthy subjects after SMF exposure (0.5 T) compared to those of non-treated cells (*p* = 0.038). The zeta potential of erythrocytes from healthy subjects (ζ = −29.9 mV) exposed to SMF (0.5 T) and to (2.0 T) was significantly decreased in comparison with the values of the electrokinetic potential of non-treated erythrocytes (ζ varied from −25.9 mV under SMF (0.5 T) exposure of up to −26.5 mV at the SMF (2.0 T) treatment) (Figure 3A). There was a significant increase in the electrokinetic potential of erythrocyte membranes from healthy subjects upon exposure to SMF (0.1 T, *p* < 0.001; 1.0 T, *p* = 0.007, and 1.5 T, *p* < 0.001) (Figure 3A). Figure 2A shows the increase in EPM (*p* = 0.014) and net surface charges of erythrocyte membranes from healthy subjects (Figure 4A) after SMF (1.5 T) treatment.

No statistically significant changes were observed in the electrophoretic mobility of β-thalassemia erythrocytes under the influence of applied SMF intensities over the entire range of exposures used (Figure 2B). SMF exposure (0.1 T; 2.0 T) led to a significant reduction in the electrokinetic potential of β-thalassemia erythrocytes (Figure 3B, *p* < 0.001). A decrease in ζ under SMF (1.0 T, 1.5 T) exposure was observed (Figure 3B, *p* = 0.011) compared to the control values of non-SMF-treated β-thalassemia erythrocytes (ζ = −23.8 mV).

However, there was a significant difference in the surface electrical charge of erythrocyte membranes from healthy subjects and β-thalassemia patients (*p* < 0.05) (Figures 4A, B). There was a reduction in the σ of non-treated erythrocytes in norm after SMF (0.5 T) exposure (σ = −0.0273 Cm^−2^) and SMF (2.0 T) (σ = −0.0281 Cm^−2^) compared to the surface charge of non-treated erythrocytes, called controls (σ = −0.0320 Cm^−2^) (*p* < 0.05) (Figure 4A). An increase of σ values of erythrocytes in norm after SMF (0.1 T; 1.5 T) treatment (*p* < 0.05) is observed.

A decrease in the negative electrical charges on the outer surface of β-thalassemia membranes was observed after SMF (0.1–2.0 T) treatment (*p* < 0.05) (Figure 4B). Net surface charge varies from σ of control samples without SMF exposure (σ = −0.0232 Cm^−2^) to σ = −0.0232 Cm^−2^ for SMF (0.1 T) treatment or σ = −0.0208 C m^-2^ at SMF (2.0 T) (*p* < 0.05). A slight decrease in negative electrical charges on the outer surface of β-thalassemia membranes at SMF (0.5 and 1.5 T) was observed (*p* < 0.05) (Figure 4B).

### 3.2 Effects of SMF on the electrostatic free energy of erythrocytes

**FIGURE 5:**
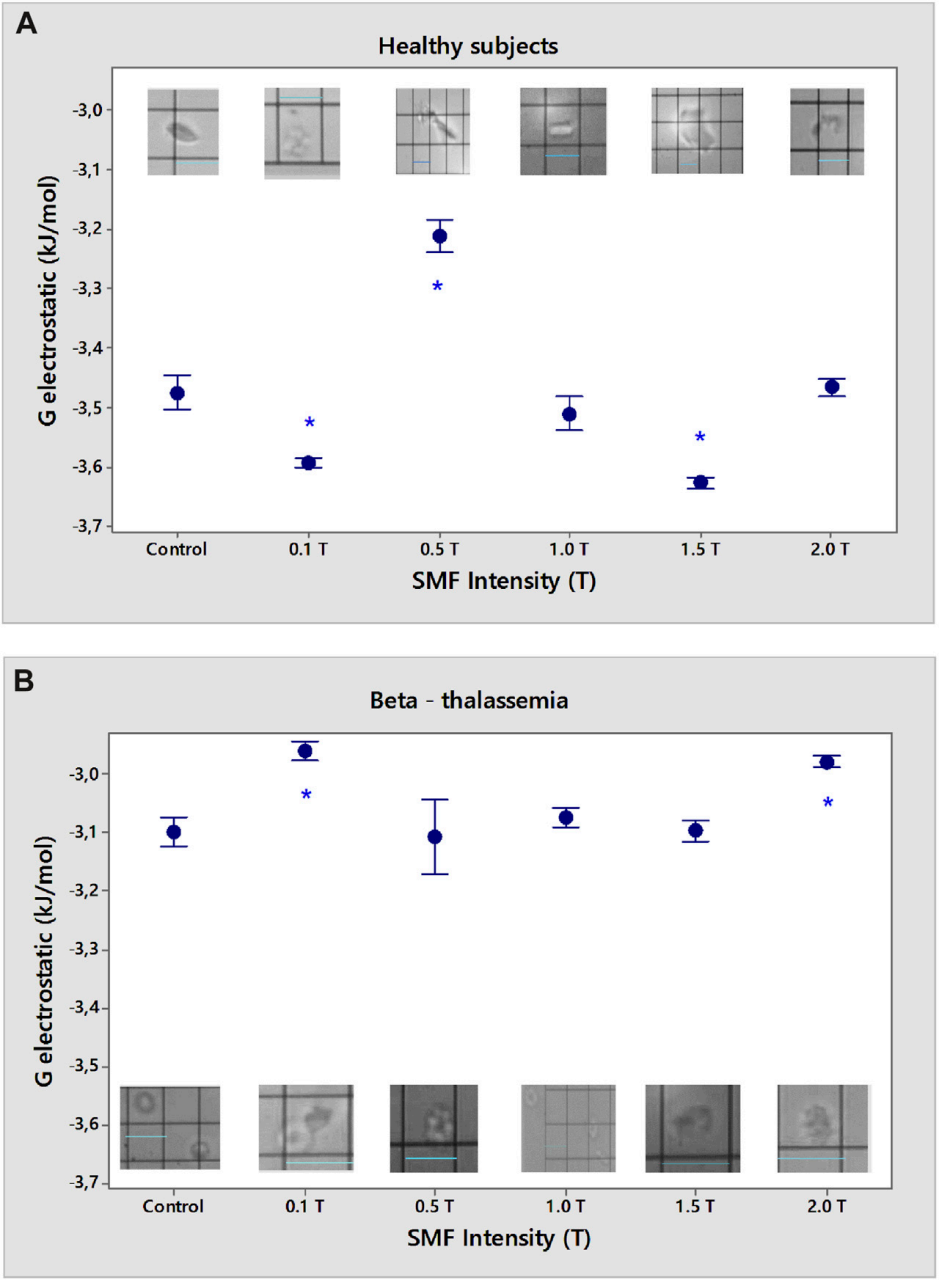
Electrostatic free energy (G_electrostatic_) of erythrocytes from healthy subjects **(A)** and β-thalassemia **(B)** as a function of different intensities of SMF in the phosphate buffered saline, pH 7.4. *p < 0.05 compared to non-treated control. Light microscopy images during microscopic (visual) microelectrophoresis (OPTON Cytopherometer) of erythrocytes in norm **(A)** and β-thalassemia **(B)** upon different intensities of SMF treatment; original magnification × 2,000, scale bar 16 µm.

Our research shows that the action of 0.1 and 0.5 T SMF causes an increase in the negative values ​​of the electrostatic free energy (G_electrostatic_), which corresponds to spontaneous processes as a result of the impact of the magnetic field on healthy erythrocytes (*p* < 0.05, Figure 5A), in contrast to the strong reduction of G_electrostatic_ at doses of 0.5 T. Lower negative values ​​of the electrostatic free energy of beta-thalassemia erythrocytes after exposure to SMF were calculated compared to the observed values ​​of G_electrostatic_ from healthy cells (*p* < 0.05, Figure 5B). The presence of spontaneous chemical processes in beta-thalassemia erythrocytes after exposure to 0.1 T and 2.0 T SMF is shown in Figure 5B (*p* < 0.05).

Shape changes are accompanied by a significant lowering of the electrostatic energy and arise in the surface area of the cell (Jadhao et al., 2014). Normally, erythrocytes have the shape of a double-concave disk, which is also visible in the microelectrophoretic microscopic measurements of the electric field of the cells. Erythrocytes treated under the norm with 0.1 T SMF are characterized by the appearance of small vesicles, and these particles are assumed to have detached from the cell membrane with the formation of vesicles according to the thermodynamic principle of maintaining maximum stability of the particle. Aggregated cells of smaller sizes resembling “cluster” aggregates are also observed (Figure 5A). SMF (0.5 T) leads to a decrease in the stability of erythrocytes in norm as a result of the decreased electrokinetic potential of the cells. Strong aggregation was observed because of the reduced zeta potential, with cells present in a larger area encompassing several cells. SMF (1.0 T) leads to the fragmentation of the erythrocyte membrane and a change in cell morphology, the serrated ends of which are well expressed on the outer surface of cells under physiological conditions. SMF (1.5 T) causes the appearance of smaller erythrocyte vesicles and the aggregation of at least two smaller cells compared to an erythrocyte in norm. We assumed that these small vesicles detached from the erythrocyte membrane because of the applied impact with the (1.5 T) SMF. Treatment of erythrocytes with SMF (2.0 T) led to the appearance of an increased number of aggregates with electrokinetic potentials close to those of healthy erythrocytes not exposed to SMF (2.0 T). There is also a fraction of erythrocytes in the norm with a smaller volume that sediments slowly when moving in an electric field and has a significantly lower zeta potential because of the lower value of the surface electric charge when treated with (2.0 T) SMF.

The β-thalassemia erythrocytes are characterized by a target-like cell shape and are larger than healthy erythrocytes. The membrane surfaces around the center of the cell (i.e., the center of the target) were slightly protruding and not convex, as was the case when the erythrocytes were exposed to certain SMF intensities (Figure 5B). When exposed to β-thalassemia erythrocytes with (0.1 T) SMF, swollen cells with protruding ends of the membrane above the main plane of the erythrocyte cell are observed. There was a change in the morphology of erythrocytes treated with (0.1 T) SMF, which were elongated along the main plane, where erythrocytes with protrusions on their surfaces were observed. SMF (0.5 T) exposure changed the erythrocyte shape. A change in the morphology of the β-thalassemia erythrocytes and reorientation and oscillation of the cells during their movement in the electric field was observed in EPM measurements after the physical impact. The target-like cells are large and swollen at their ends. Erythrocytes may have an elongated shape compared to β-thalassemia control erythrocytes, with a reduced cell target area. Treatment of β-thalassemia erythrocyte membranes with an SMF (1.0 T) caused a change in erythrocyte morphology. The cells have a smaller concave portion and highly convex membrane surfaces. A jagged outer cell envelope is also observed. SMF (1.5 T) also changed the shape of β-thalassemia erythrocytes, with protrusions above the plane of the outer membrane of the cell. Thus, changes in the morphology of β-thalassemia cells were caused by protrusions above the membrane surface. Erythrocytes isolated from patients with β-thalassemia treated with 1.5 T SMF move in the electric field by changing the direction of their movement from horizontal (in the direction of the opposite pole of the negatively charged cell) to the vertical position that appeared upright in their movement (occupying a perpendicular position relative to the plane of the electrophoretic chamber). β-thalassemia erythrocytes, after being treated to (2.0 T) SMF, have a changed cell morphology. Swollen “target-like cells” have been recorded, which appear swollen around the erythrocyte cavity with a smaller area. The shape change of β-thalassemia erythrocytes possesses protrusions above the membrane, protruding swollen ends of the plasma membrane of the cell, and cells with larger sizes of the “target-like” β-thalassemia erythrocytes.

### 3.3 Effects of SMF on the light scattering of erythrocytes

The level of light scattering (LS) at a 90° angle with respect to the incident beam direction has been used to monitor erythrocyte aggregation after SMF exposure (Doltchinkova et al., 2006).

**FIGURE 6:**
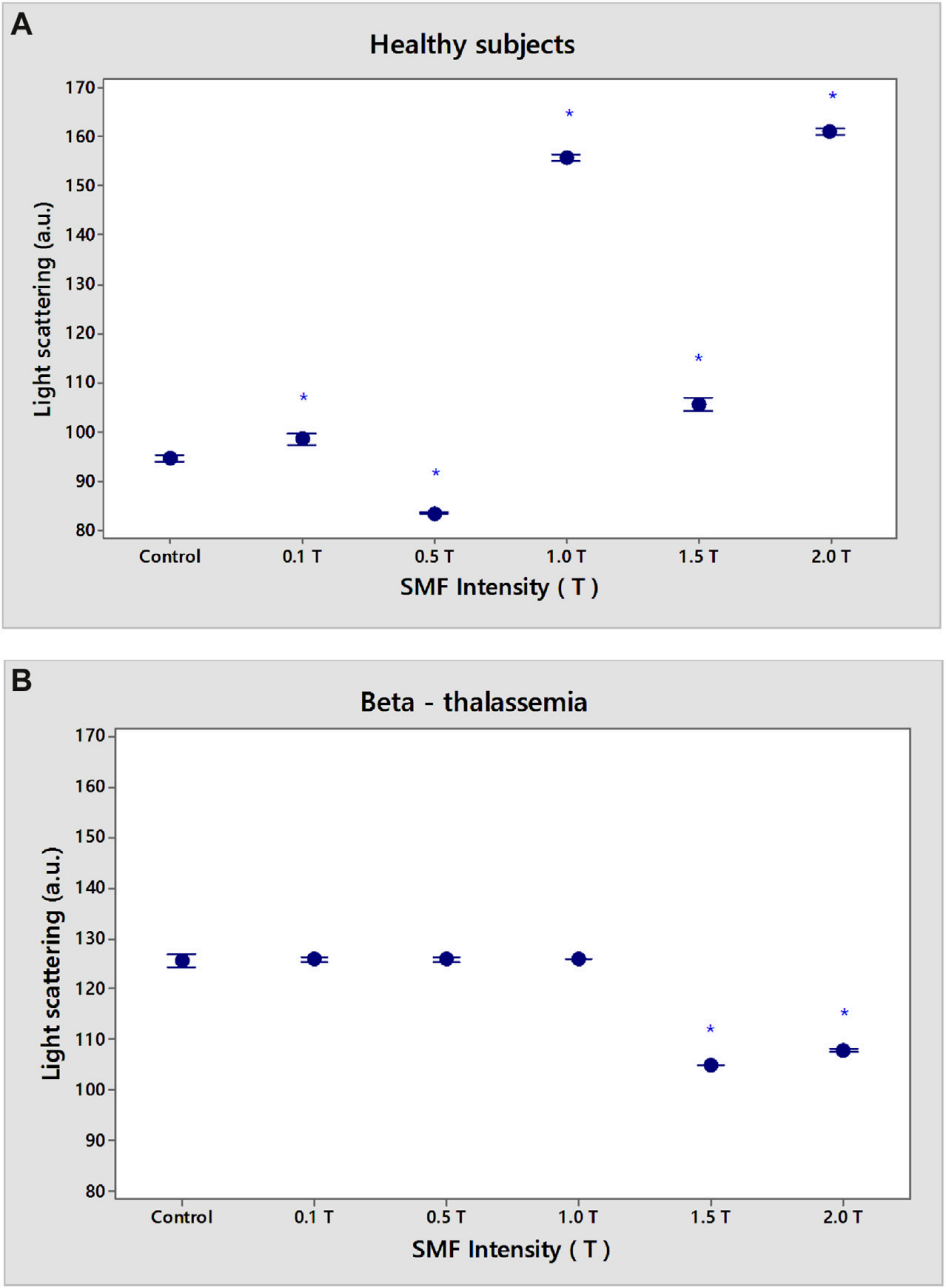
Effect of static magnetic field (0.1–2.0 T) exposure on light scattering of erythrocyte membranes in norm **(A)** and β-thalassemia **(B)**. The solution contains phosphate buffered saline (PBS), pH 7.4. Data are the means (vertical bars indicate SD) of three independent experiments, each of three replications. In the LS experiment with erythrocyte membranes, the sample is measured at 25°C through KC 13 red and blue-green glass filters. An amplitude resolution equivalent to an intensity change ∆I/I = 1 × 10^−9^ is used in the LS measurement. *p < 0.05 Control value without SMF exposure compared to SMF treatment of erythrocytes from healthy subjects **(A)**; *p < 0.05 Control value without SMF exposure compared to SMF treatment of erythrocytes from β-thalassemia patients **(B)**.

The exposure of erythrocytes from healthy subjects to SMF is shown in Figure 6A. The LS of erythrocytes was reduced following the treatment with SMF (0.5 T). The intensities of the SMF (0.1 T, 1.0–2.0 T) significantly enhanced the LS value (*p* < 0.05). There was a significant decrease in the LS of β-thalassemia erythrocytes in SMF (1.5 T and 2.0 T) (*p* < 0.05) in comparison to that of control erythrocytes in pathology without SMF treatment (Figure 6B).

### 3.4 Lipid peroxidation: Detection of malondialdehyde (MDA) content

The extent of lipid peroxidation (LP) was estimated by analyzing thiobarbituric acid reactive substances (TBARS) according to the method described by Halliwell and Gutteridge (1999) with modifications.

Magnetic fields are one of the most common environmental factors that influence living systems by increasing the lifespan of free radicals. Magnetic fields influence the kinetics of reactions with radical pair intermediates (McLauchlan and Steiner, 1991; Grissom, 1995). We have studied: i) whether SMF (0.1–2.0 T) induces lipid peroxidation stress in erythrocytes in normal and pathological conditions and ii) compared the lipid peroxidation status of erythrocytes in the presence of 50 mM H_2_O_2_ (second positive control), where the maximal LP changes of erythrocytes in normal conditions are observed.

**FIGURE 7:**
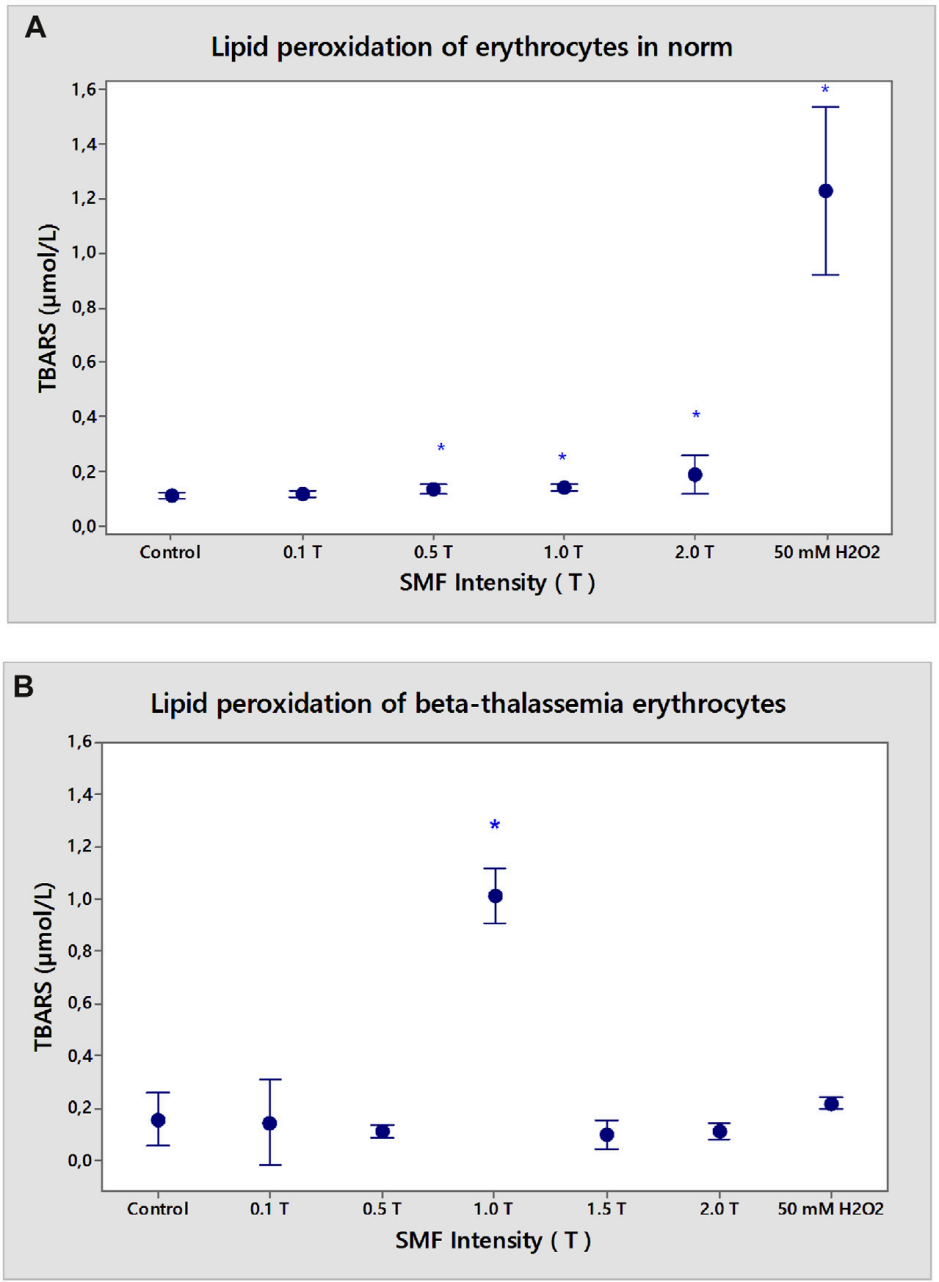
Effect of static magnetic field (0.1–2.0 T) on lipid peroxidation of erythrocyte membranes in norm **(A)** and β-thalassemia **(B)**. Influence of static magnetic field on lipid peroxidation of erythrocyte membranes in norm and β-thalassemia suspended in phosphate buffered saline, pH 7.4. Malondialdehyde content in the erythrocyte membranes, isolated from healthy subjects and β-thalassemia patients in physiological medium (PBS, pH 7.4). Effect of SMF (0.1–2.0 T) pre-exposure on erythrocytes after incubation in phosphate buffered saline (PBS), pH 7.4 for 1 h at 37°C. MDA is determined by the supernatant, as described in the text. Values are expressed as TBARS after SMF pre-exposed erythrocyte membrane values compared to TBARS of non-treated erythrocytes without SMF (*n* = 3). *p < 0.05 compared to non-treated control. The positive control of 50 mM H_2_O_2_ represents the maximal value of TBARS products (incubation of erythrocytes in the presence of 50 mM H_2_O_2_ for 1 h at 37°C).

The content of MDA, a marker of lipid peroxidation stress, was examined in SMF (2.0 T)-pre-exposed erythrocytes (Figures 7A, B). In an isotonic solution, the lipid peroxidation rate of erythrocytes under normal conditions increased upon adding 50 mM H_2_O_2_ (Figure 7A). The SMF exposure promotes the lipid peroxidation of erythrocytes from healthy subjects at doses of SMF (0.5–2.0 T), compared to the control SMF-non-treated cells (Figure 7A). SMF (1.0 T) treatment of β-thalassemia erythrocytes enhanced secondary LP products in the cells (*p* < 0.05).

### 3.5 Fluorescence microscopy of erythrocyte membranes in norm and pathology

**FIGURE 8:**
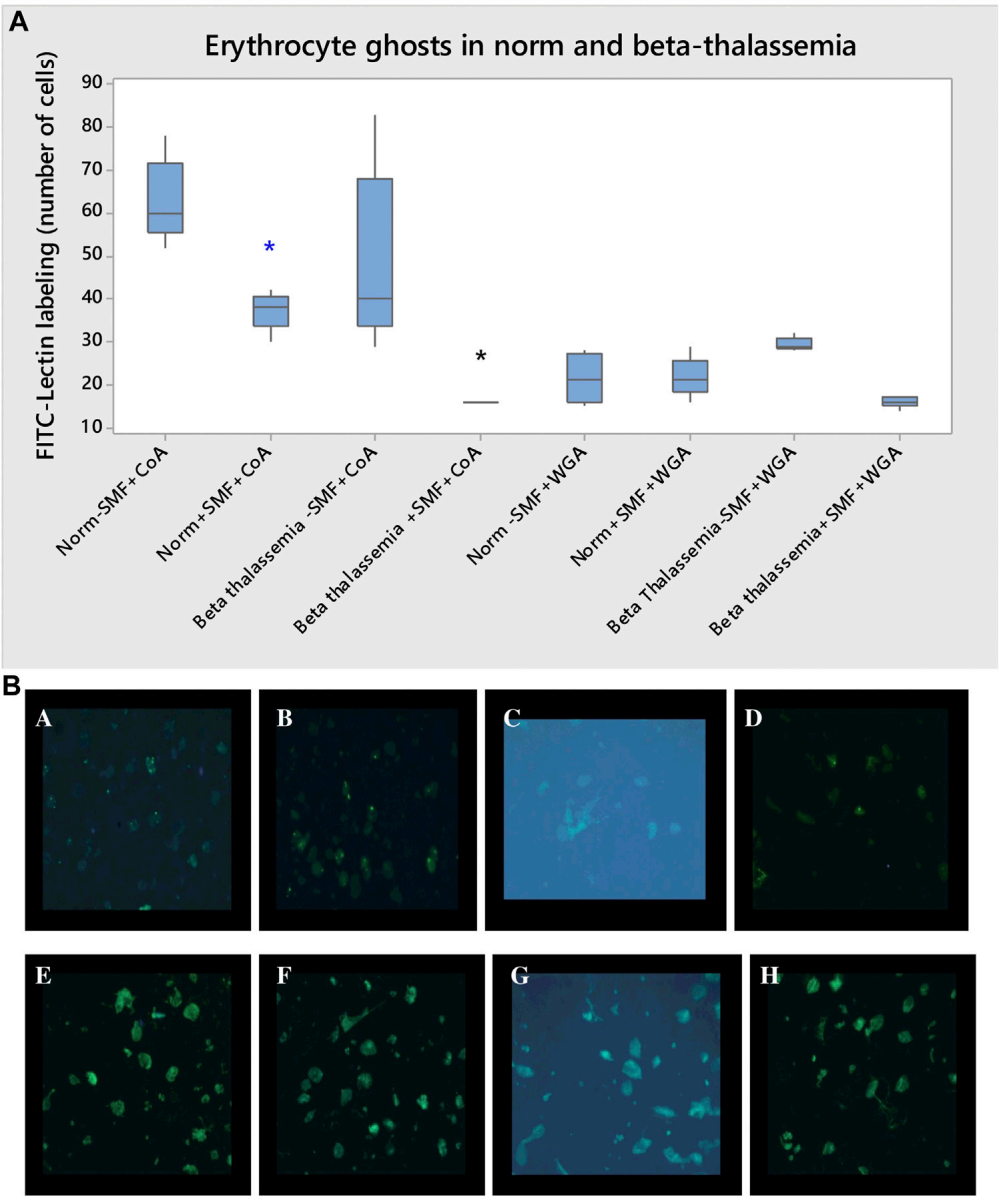
**(A)** FITC-lectin labeling of erythrocyte membranes (erythrocyte ghosts) in norm and β-thalassemia after SMF (2.0 T) treatment. FITC-concanavalin A (FITC-CoA) and FITC-wheat germ agglutinin (FITC-WGA) labeling of erythrocyte membranes in norm and β-thalassemia after SMF (2.0 T) treatment. The One-Way ANOVA test for norm- and pathology data is performed. The differences among the means are significant (*p < 0.05). Each value is the mean ± SD of three independent preparations with three repetitions each. *p < 0.05 compared to FITC-CoA-containing control in norm (blue asterisk) or FITC-CoA-containing control in β-thalassemia (black asterisk). **(B)** Images of FITC-lectin labeling of human erythrocyte ghosts from healthy subjects and patients upon exposure to SMF (2.0 T). Effect of FITC-concanavalin A (FITC-CoA) on erythrocyte ghosts from healthy subjects without SMF (2.0 T) treatment. Effect of FITC-concanavalin A (FITC-CoA) on erythrocyte ghosts from healthy subjects after SMF (2.0 T) treatment. Effect of FITC-concanavalin A (FITC-CoA) on erythrocyte ghosts from β-thalassemia without SMF (2.0 T) treatment. Effect of FITC-concanavalin A (FITC-CoA) on erythrocyte ghosts from β-thalassemia upon SMF (2.0 T) treatment. Effect of FITC-wheat germ agglutinin (FITC-WGA) on erythrocyte ghosts from healthy subjects before SMF (2.0 T) treatment. Effect of FITC-wheat germ agglutinin (FITC-WGA) on erythrocyte ghosts from healthy subjects after SMF (2.0 T). Effect of FITC-wheat germ agglutinin (FITC-WGA) on erythrocyte ghosts from β-thalassemia patients before SMF (2.0 T) treatment. Effect of FITC-wheat germ agglutinin (FITC-WGA) on erythrocyte ghosts from β-thalassemia patients after SMF (2.0 T) exposure.

Erythrocyte ghosts prepared by lysis and washed with hypotonic phosphate buffer had the same lipid content as intact red blood cells. Figure 8A shows the changes in FITC-lectin labeling in the epifluorescence (expressed as the amount of FITC-lectin labeling of the cells) in erythrocyte membranes (erythrocyte ghosts) before and after SMF (2.0 T) treatment.

Healthy erythrocyte membranes treated with FITC- concanavalin A (FITC-CoA) and FITC-wheat germ agglutinin (FITC-WGA) are presented with intense fluorescence due to lectins binding to the membrane (Figure 8A). Weaker fluorescence resulting from lectin-labeling of treated erythrocyte membranes from healthy subjects was observed after exposure to SMF (2.0 T) (Figure 8A) (*p* < 0.05). The binding of FITC-CoA to thalassemia erythrocyte membranes was reduced compared to that in non-treated ghosts in pathology without SMF (2.0 T).

Fluorescent microscopy images of FITC-CoA and FITC-WGA labeling of erythrocyte membranes after SMF (2.0 T) treatment in normal and β-thalassemia are presented in Figure 8B. The erythrocyte model systems can be further utilized to obtain information on protein-membrane interactions under SMF (2.0 T) exposure.

## 4 Discussion

The static magnetic field has been proven to influence the surface electrical characteristics of erythrocyte membranes under normal and pathological conditions. Treatment of healthy erythrocytes at an intensity of SMF (0.1 T) leads to an increase in zeta potential and surface electrical charge of the cells compared to untreated cells. The upper SMF treatment caused an increase in light scattering (i.e., cell shrinkage). The opposite effect is observed in the treatment of β-thalassemia erythrocytes. β-thalassemia erythrocytes treated with SMF (0.1 T) showed reduced electrokinetic potential and surface electrical charge compared to non-treated cells. Decreases in zeta potential values of SMF-treated β-thalassemia erythrocytes suggest a decrease in electrostatic repulsion forces between cells and a relative increase in hydrophobic and local Van der Waals forces between cells in close proximity. The formation of LP products from erythrocytes was not observed at the normal and pathological levels after exposure to SMF (0.1 T).

SMF (0.5 T) treatment leads to a decrease in their EPM, ζ potential, and surface electric charge, in contrast to the opposite effect in σ of β-thalassemia erythrocytes. There was a decrease in the LS (an increase in cell volume) of erythrocytes after SMF (0.5 T) exposure. Enhanced values of lipid peroxidation in erythrocytes from healthy subjects were found, in contrast to the lack of changes in the LP of β-thalassemia erythrocytes after SMF (0.5 T) treatment.

Exposed to SMF (1.0 T), erythrocytes are characterized by an increase in ζ potential and σ of cells in norm compared to non-treated ones. A decreased zeta potential due to a reduction in the surface charge of erythrocytes from β-thalassemia was observed after treatment with SMF (1.0 T). An increase in the LS of erythrocyte suspensions has been reported in norm compared to non-treated ones. Statistically significant changes were observed in the LP of erythrocytes after exposure to SMF (0.5‒2 T). Exposure to SMF (1.0 T) alone caused an increase in TBARS levels compared to unexposed β-thalassemia cells.

An increase in EPM, ζ potential, σ, and LS (decrease in cell volume) is observed after the SMF (1.5 T) treatment of healthy erythrocytes compared to non-treated cells. No changes were observed in the formation of secondary products of LP in erythrocytes after treatment with SMF (1.5 T). A decrease in the electrokinetic potential and σ of β-thalassemia erythrocytes after treatment with SMF (1.5 T) is observed.

SMF (2.0 T) treatment of healthy or β-thalassemia erythrocytes affects the ζ potential, leading to its reduction because of the decrease in the net surface electrical charges exposed on the outer surface of the membrane compared with that of unexposed cells.

The rate and size of erythrocyte aggregation can be used as biomarkers for many clinical conditions and are vital signs of diseases, such as diabetes (Ami et al., 2001) and deep thrombosis (Yu et al., 2011). LS techniques are used to measure erythrocyte membrane aggregation (Dybas et al., 2022). SMF can modify the mechanisms of erythrocyte aggregation (Doltchinkova et al., 2006; Elblbesy, 2017). Erythrocyte swelling of β-thalassemia (Pribush et al., 2003) compared to LS level of cells from healthy subjects changes the aggregation of erythrocytes in pathology. The effects of SMF (2.0 T) exposure on erythrocytes under normal and β-thalassemia conditions were characterized by the opposite effects of LS changes. SMF (2.0 T) treatment led to an increase in the LS of erythrocytes from healthy subjects and a decrease in β-thalassemia erythrocytes compared to non-treated erythrocytes under normal and pathological conditions. Thus, changes in volume and cell morphology can be the primary factors in clarifying the influence of SMF (2.0 T) on erythrocytes in norm and β-thalassemia. One of the main results is the fact that SMF action on the electrokinetics of the erythrocyte membrane is a spontaneous chemical process. Electrostatic free energy is characterized by a change in negative G_electrostatic_ values under SMF treatment with different intensities. As the magnitude of negative change in G_electrostatic_ becomes greater, so goes the tightness of concanavalin A binding or increase in CoA binding affinity properties of the SMF action on the erythrocyte membranes compared to non-treated ones.

SMFs (≥100 µT) enhance the reactive oxygen species (ROS) levels (Calabrò et al., 2013; Vergallo et al., 2014; Zablotskii et al., 2014; Wang and Zhang, 2017); however, the mechanism by which magnetic field exposure modulates ROS concentration in the cells still remains unclear. Weak magnetic fields can change free-radical reactions and concentrations in biological systems and influence specific cellular functions (Barnes and Greenebaum, 2015; Montoya, 2017). The presented results show that SMF (0.5–2.0 T) could intensify a generation of reactive oxygen species with disturbances of LP processes (i.e., secondary products of LP) in erythrocytes from healthy subjects. There was an increase in TBARS products in β-thalassemia erythrocytes upon SMF (1.0 T) treatment. SMF (2.0 T)-exposed samples were significantly different from the positive control in the presence of 50 mM H_2_O_2,_ where the maximal increase in the LP of erythrocytes under normal conditions is expected.

SMF (2.0 T) exposure induced a decrease in FITC-concanavalin A binding to erythrocyte membranes in the normal and β-thalassemia groups compared with the control values of non-treated membranes. SMF (2.0 T) leads to a decrease in the electrokinetic potential and the binding of FITC-lectin to the membrane because of the restricted binding to the lectin receptors on the outer surface of the membrane by erythrocytes in norm and β-thalassemia. The increased aggregation of erythrocytes under normal conditions is associated with decreased erythrocyte volume in the complex and an increase in the area of the aggregated erythrocyte complex, which is accompanied by increased lipid peroxidation under the influence of an SMF (2.0 T). The decreased aggregate formation is not associated with the formation of TBARS products during LP of β-thalassemia erythrocyte membranes. There was a lower binding of FITC-WGA compared to FITC-CoA to β-thalassemia erythrocyte ghosts due to stronger inhibition of lectin interactions with membrane receptors in pathology under the influence of SMF (2.0 T).

At present, the biological processes causing SMF alteration in the electrokinetic potential must be explained. It is well known that the application of magnetic fields causes the orientation of biomolecules and biopolymers owing to their diamagnetic isotropy (Portelli, 2019). Magnetic field effects have been useful for verifying the mechanism of the electrokinetic and LS properties of erythrocytes in norm and β-thalassemia.

## 5 Conclusion

The data received show that biophysical properties of erythrocytes in norm and pathology (β-thalassemia) vary with exposure to SMF (0.1–2.0 T). The effect of static magnetic field (SMF 0.1–2.0 T) on the electrokinetic and morphological characteristics of erythrocytes in norm and β-thalassemia is determined and correlated with the increase/reduction in surface charge and shrinkage/swelling of the cells, respectively. Lipid peroxidation of healthy and β-thalassemia erythrocytes caused an enhancement of lipid peroxidation because of the higher concentrations of TBARS products in cellular suspension. Influence of FITC-Concanavalin A and FITC-Wheat Germ Agglutinin binding was tested for both types of erythrocyte membranes upon SMF (2.0 T) treatment. SMF (0.1–2.0 T) altered the spontaneous chemical processes with negative values of electrostatic free energy of erythrocytes in norm and β-thalassemia accompanied by a lower FITC-Concanavalin A binding affinity to membrane receptors (SMF 2.0 T). The electrokinetic properties of human erythrocytes in norm and β-thalassemia upon SMF treatment and their interrelationship with the structural-functional state of the membrane were reported. The presented work would have future fundamental applications in biomedicine.

## Data Availability

The original contributions presented in the study are included in the article/Supplementary Material, further inquiries can be directed to the corresponding author.

## Abbreviations

CoA, concanavalin A; DAPI, (4′,6-Diamidino-2′-phenylindole dihydrochloride); DDL, diffuse double layer; EPM, electrophoretic mobility; FITC, Fluorescein isothiocyanate; G_electrostatic_, electrostatic free energy; LP, lipid peroxidation; LS, light scattering; MDA, malondialdehyde; PBS, phosphate buffered saline; PMSF, Phenylmethanesulfonyl fluoride; SD, standard deviation; SMF, static magnetic field; T, Tesla; TBA, 2-thiobarbituric acid; TBARS, thiobarbituric acid reactive substances; TCA, trichloroacetic acid; WGA, wheat germ agglutinin; ζ potential, zeta (electrokinetic) potential; σ, surface electrical charge (surface charge density).
