# Supplementary material for: Electrokinetic properties of healthy and β-thalassemia erythrocyte membranes under in vitro exposure to static magnetic field
Source: Front Chem. 2023 Oct 19;11:1197210. doi: 10.3389/fchem.2023.1197210 (PMC10620691; doi:10.3389/fchem.2023.1197210)
Supplement: Supplementary file 1 [file DataSheet1.pdf]

Supplementary Material

Electrokinetic properties of healthy and β-thalassemia erythrocyte membranes under *in vitro* exposure to static magnetic field

Virjinia Doltchinkova*, Siya Lozanova, Blaga Rukova, Rumen Nikolov, Elitsa Ivanova, Chavdar Roumenin

*** Correspondence:** Virjinia Doltchinkova: vdoltchinkova@gmail.com

1 Supplementary Data

2 Supplementary Figures and Tables

2.1 Supplementary Figures

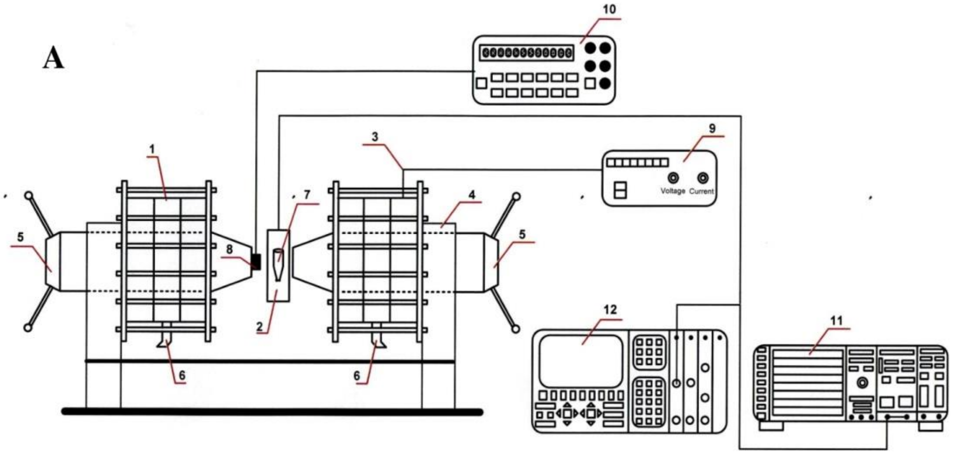

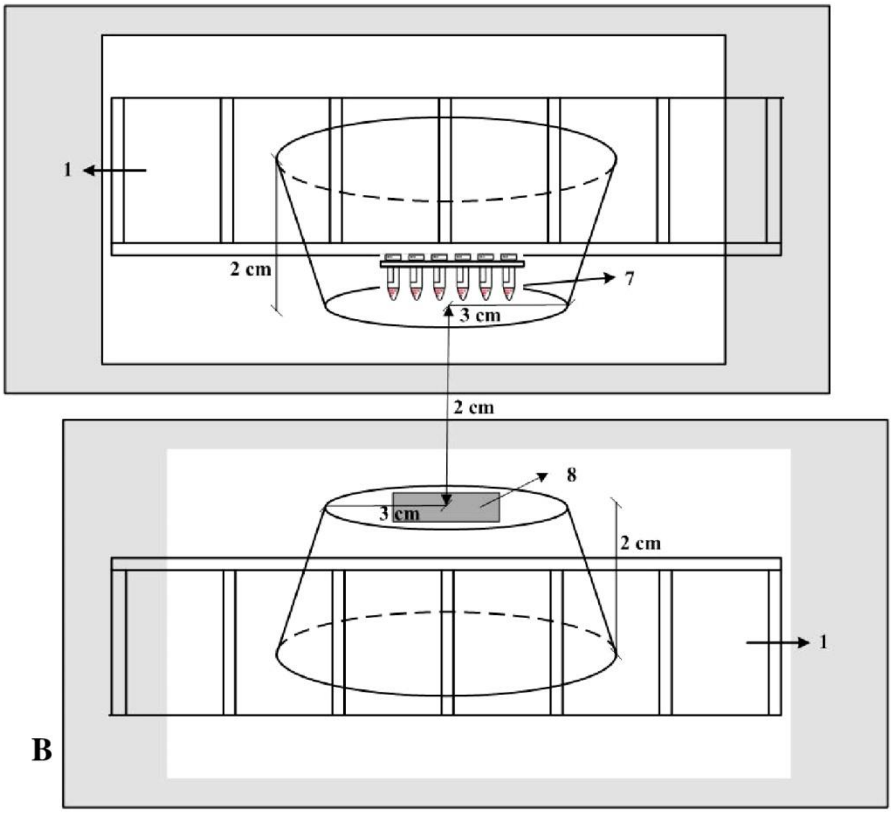

Supplementary Figure 1 (A) Experimental setup used for SMF exposure of erythrocyte samples. 1-Weiss U type electromagnet with water cooling; 2-Eppendorf tube (1.5 ml) containing the sample; 3-Constant current generator; 4-Electromagnit yoke; 5-Perpendurelectromagnetic poles (an iron mild magnetic alloy containing 48-50% of Co with magnetic induction maximum of 2.4 T), ϕ=60 mm; 6-Water cooling; 7-Samples; 8-Hall sensor type KSY 14 calibrated for determining the direction and induction field B; 9-Supply voltage block HP 34401A; 10-Digital measure block HP 34401A; 11-HP 1980B oscilloscope; 12-Philips spectral analyzer (model #PM3360). (**B)** Experimental scheme used for SMF treatment of erythrocyte samples. The erythrocyte sample tubes are placed on a Plexiglas stand with six wells in which the Eppendorf tubes are placed in a vertical position, attached to one pole of the magnet in a thermostatically adjustable chamber. 1-Weiss U type electromagnet with water cooling; 7-Samples; 8-Hall sensor type KSY 14 calibrated for determining the direction and induction field B. Radius of distance between poles (inter-pole distance) is 2.0 cm; radius of working area of truncated cone is 3.0 cm; height of truncated cone is 2.0 cm.

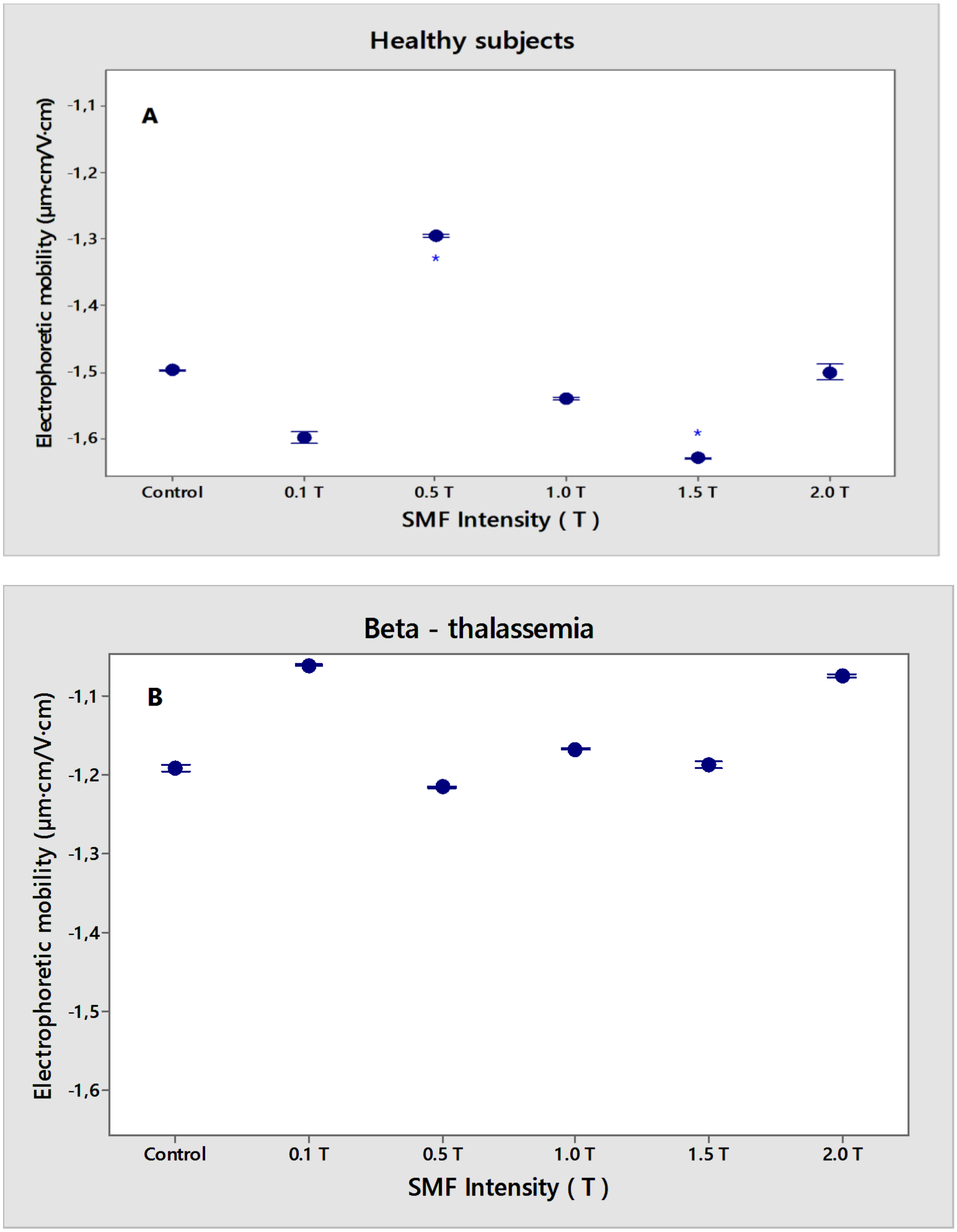

Supplementary Figure 2. Dependence of SMF (0.1 – 2.0 T) on electrophoretic mobility (EPM) of erythrocyte membranes in norm (**A**) and β-thalassemia (**B**). The solution contains phosphate buffered saline (PBS), pH 7.4. EPM of erythrocytes in norm and pathology is measured after 15 min SMF pre-exposure at 25 °C. Values are expressed as mean ± SD (n= 54 - 75) of three independent measurements, each of three replications. *$p<0.05$.

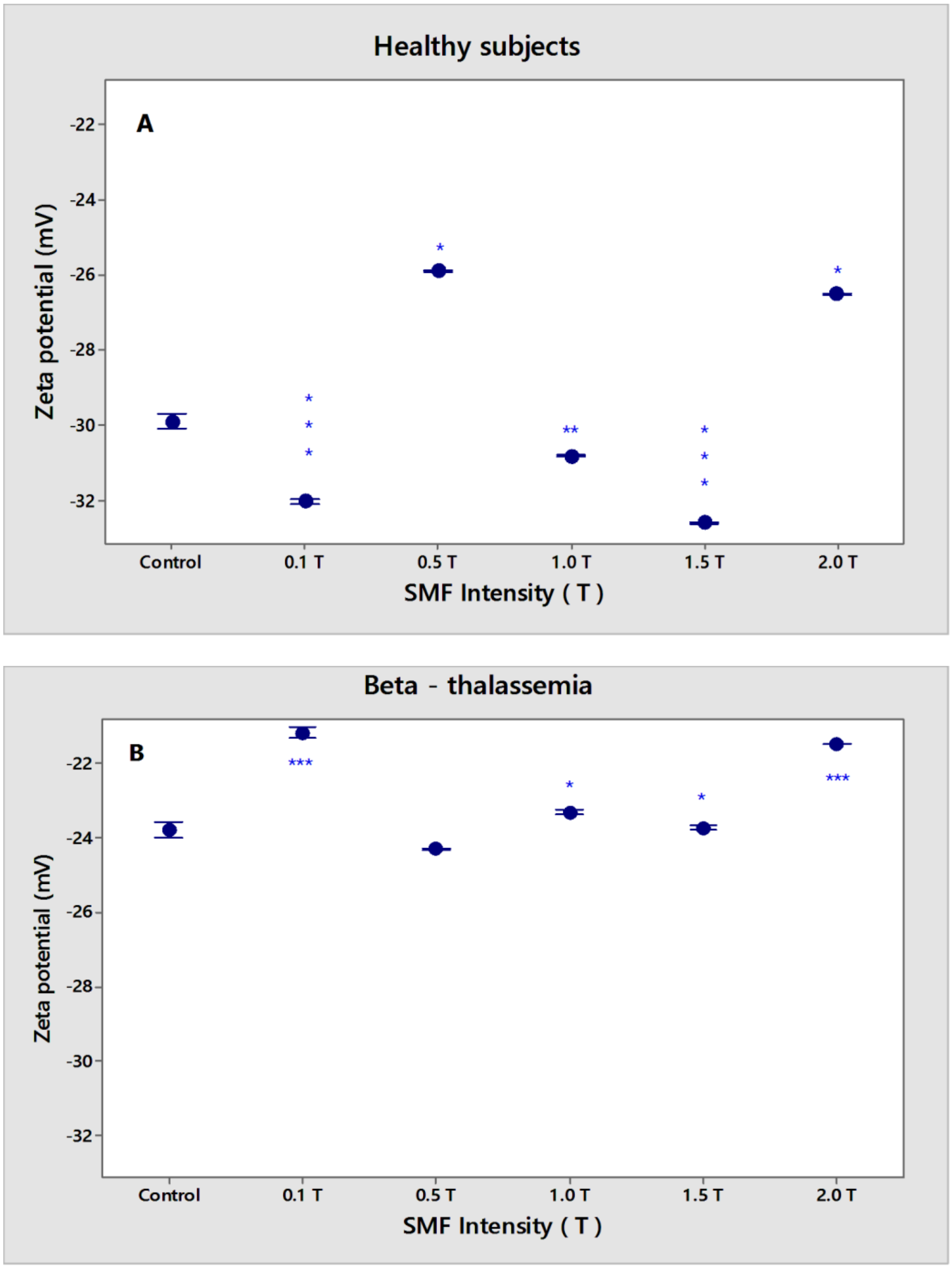

Supplementary Figure 3. Dependence of SMF (0.1 – 2.0 T) on zeta potential (ζ) of erythrocyte membranes in norm (**A**) and β-thalassemia (**B**). The solution contains phosphate buffered saline (PBS), pH 7.4. EPM of erythrocytes in norm and pathology is measured after 15 min SMF pre-exposure at 25 ^{o}C. Values are expressed as mean ± SD of three independent measurements, each of three replications. *p<0.05; **p<0.01; ***p<0.001.

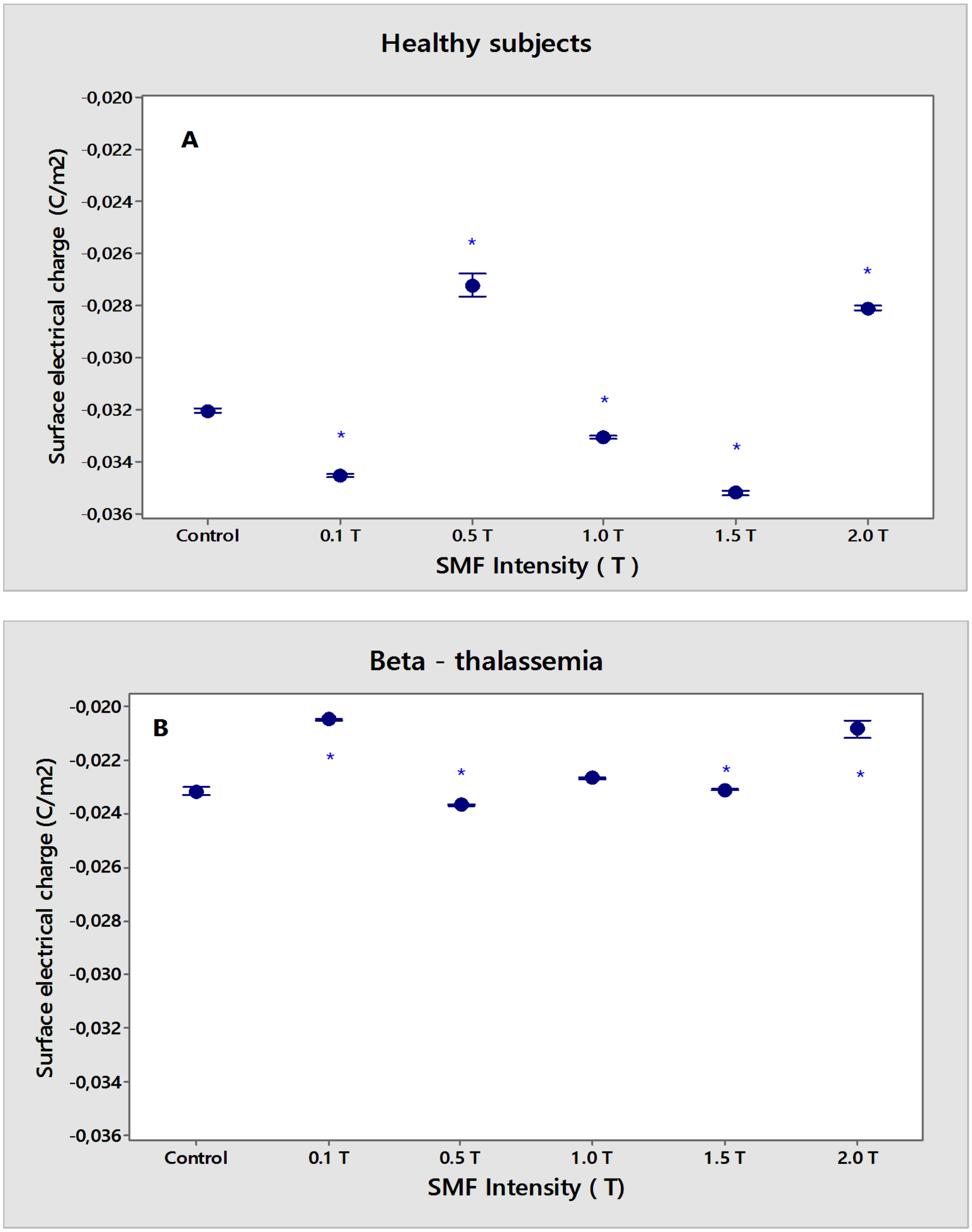

Supplementary Figure 4. Dependence of SMF (0.1 – 2.0 T) on surface electrical charge (σ) of erythrocyte membranes in norm (**A**) and β-thalassemia (**B**). The solution contains phosphate buffered saline (PBS), pH 7.4. EPM of erythrocytes in norm and pathology is measured after 15 min SMF pre-exposure at 25 ^{o}C. Values are expressed as mean ± SD of three independent measurements, each of three replications. *$p<0.05$.

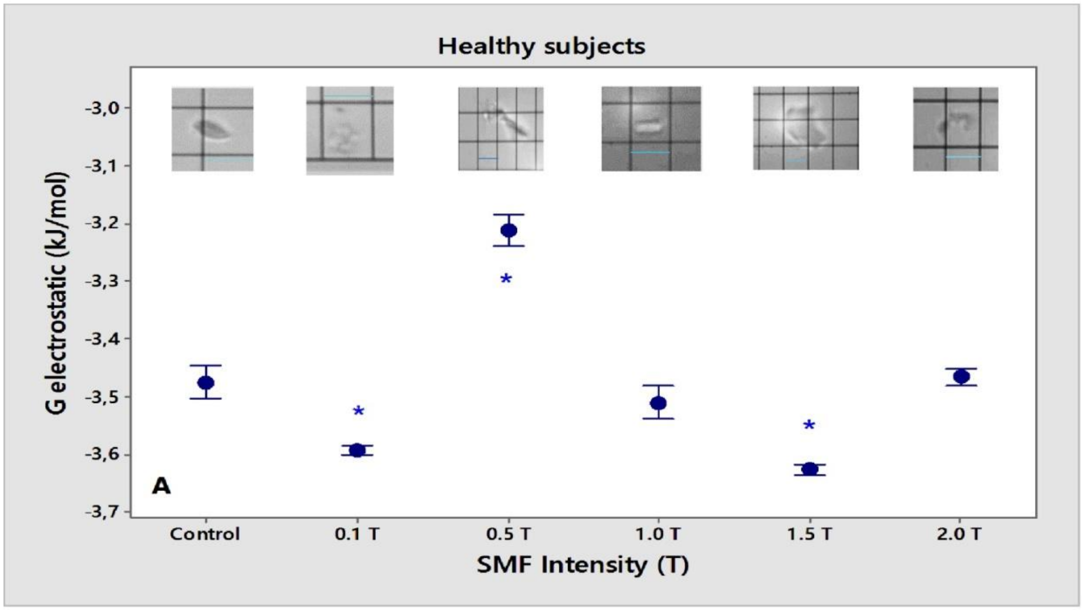

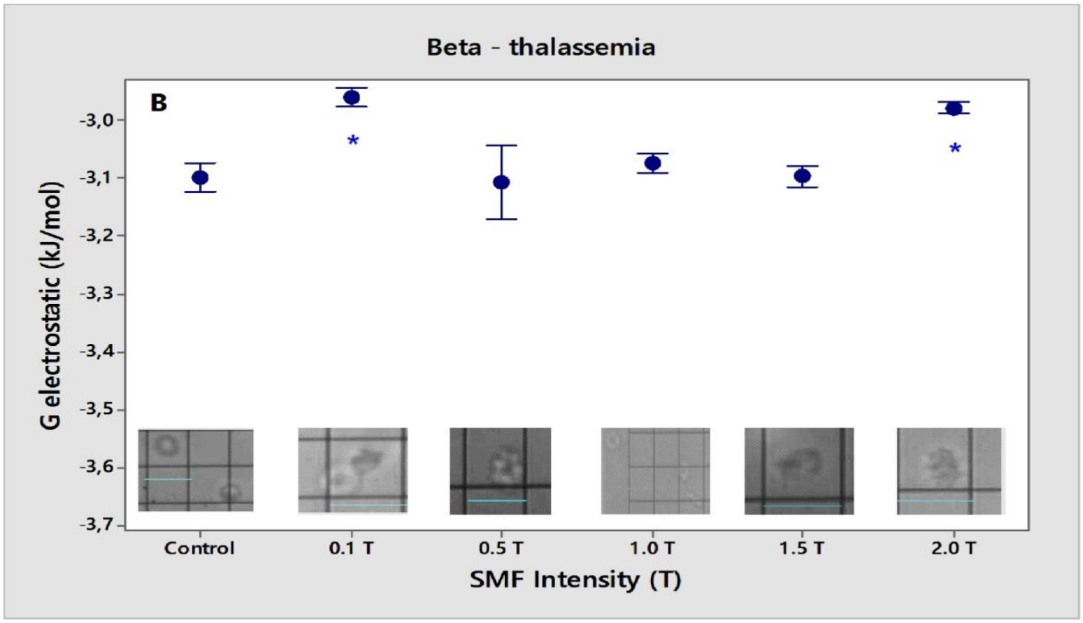

Supplementary Figure 5. Electrostatic free energy ($G_{electrostatic}$) of erythrocytes from healthy subjects (**A**) and β-thalassemia (**B**) as a function of different intensities of SMF in the phosphate buffered saline, pH 7.4. *$p<0.05$ compared to non-treated control. Light microscopy images during microscopic (visual) microelectrophoresis (OPTON Cytopherometer) of erythrocytes in norm (**A**) and β-thalassemia (**B**) upon different intensities of SMF treatment; original magnification x 2000, scale bar 16 µm.

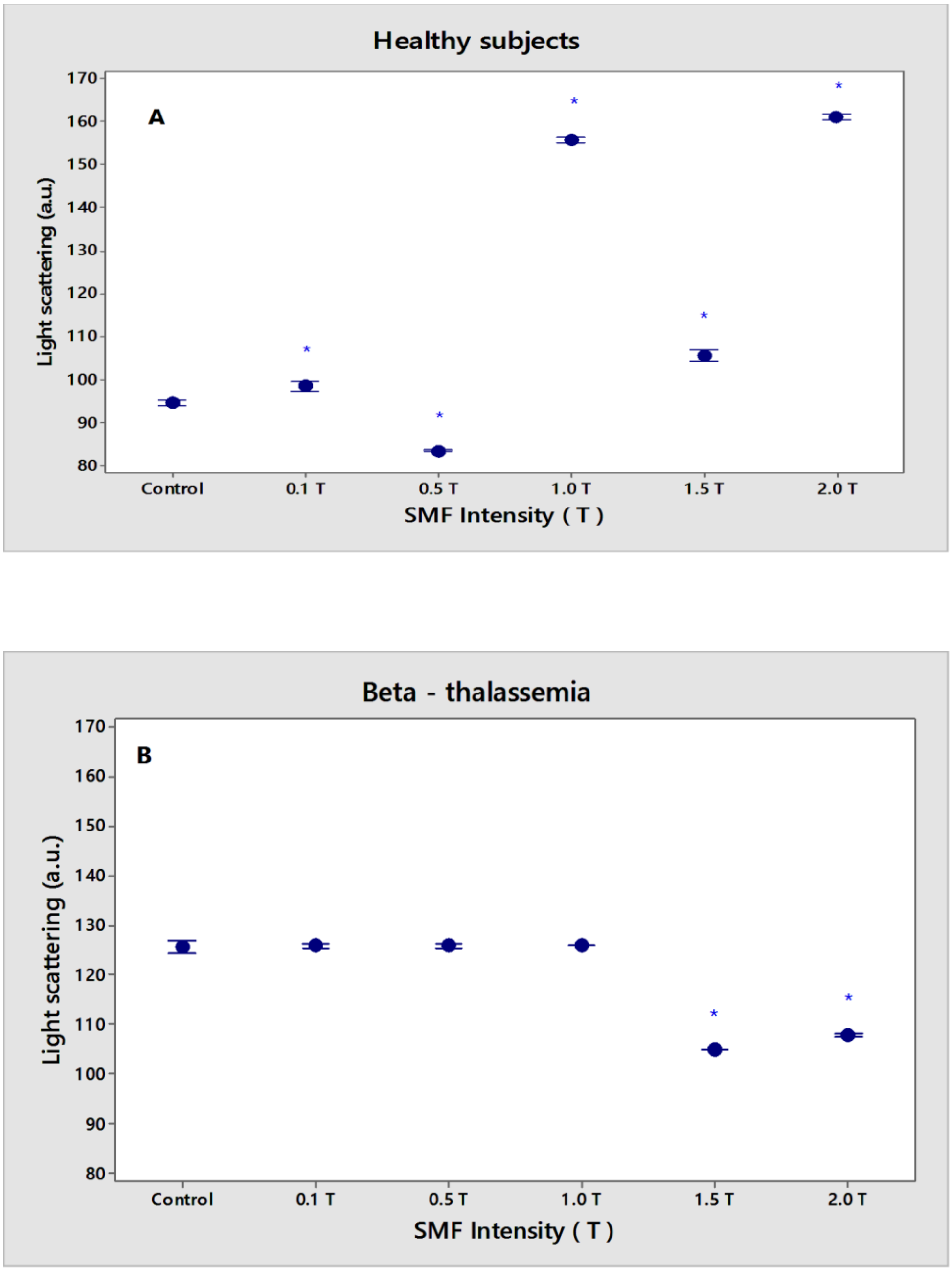

Supplementary Figure 6. Effect of static magnetic field (0.1–2.0 T) exposure on light scattering of erythrocyte membranes in norm (**A**) and β-thalassemia (**B**). The solution contains phosphate buffered saline (PBS), pH 7.4. Data are the means (vertical bars indicate SD) of three independent experiments, each of three replications. In the LS experiment with erythrocyte membranes, the sample is measured at 25 °C through KC 13 red and blue-green glass filters. An amplitude resolution equivalent to an intensity change $\Delta I/I = 1 \times 10^{-9}$ is used in the LS measurement. *p<0.05 Control value without SMF exposure compared to SMF treatment of erythrocytes from healthy subjects (**A**); *p<0.05 Control value without SMF exposure compared to SMF treatment of erythrocytes from β-thalassemia patients (**B**).

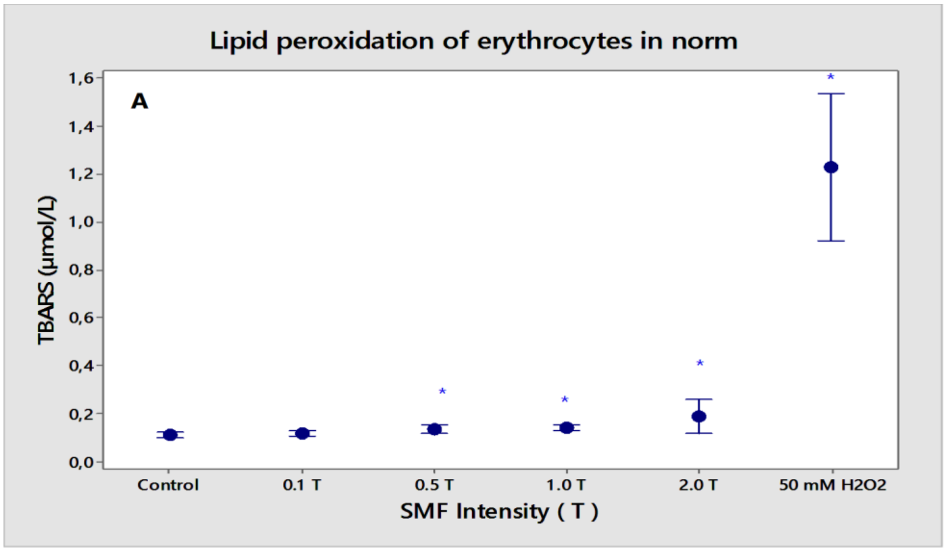

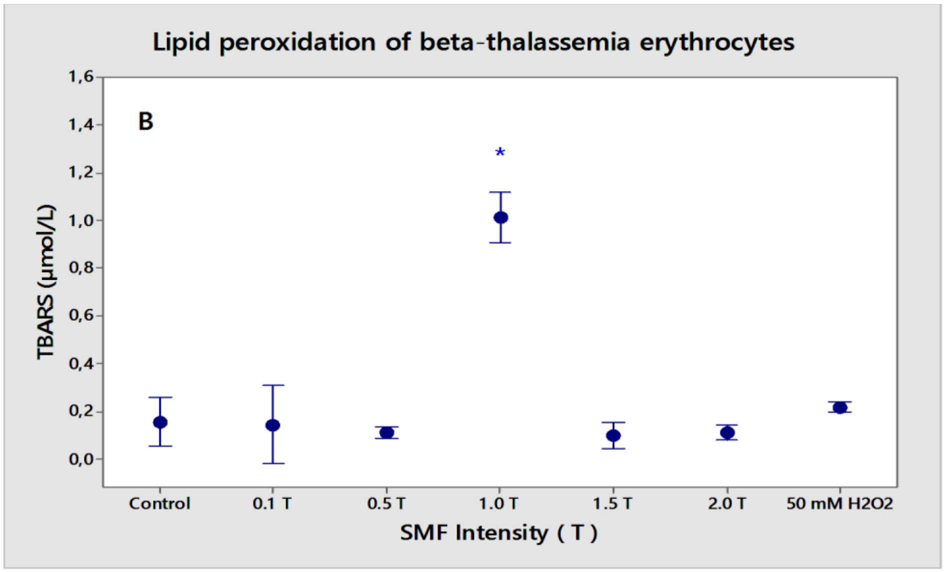

Supplementary Figure 7. Effect of static magnetic field (0.1 – 2.0 T) on lipid peroxidation of erythrocyte membranes in norm (**A**) and β-thalassemia (**B**). Influence of static magnetic field on lipid peroxidation of erythrocyte membranes in norm and β-thalassemia suspended in phosphate buffered saline, pH 7.4. Malondialdehyde content in the erythrocyte membranes, isolated from healthy subjects and β-thalassemia patients in physiological medium (PBS, pH 7.4). Effect of SMF (0.1 T – 2.0 T) pre-exposure on erythrocytes after incubation in phosphate buffered saline (PBS), pH 7.4 for 1 hour at 37 ^{o}C. MDA is determined on the supernatant as described in the text. Values are expressed as TBARS after SMF pre-exposed erythrocyte membranes values compared to TBARS of non-treated erythrocytes without SMF (n=3). Bar presents TBARS in non-exposed to SMF erythrocytes and SMF exposed samples. *p<0.05: SMF (2.0 T) compared to 50 mM H_2O_2 (**A**); *p<0.05 Control values compared to SMF (2.0 T; 0.1 T; 50 mM H_2O_2; 0.5 T and 0.1 T) after SMF treatment, respectively (**B**).

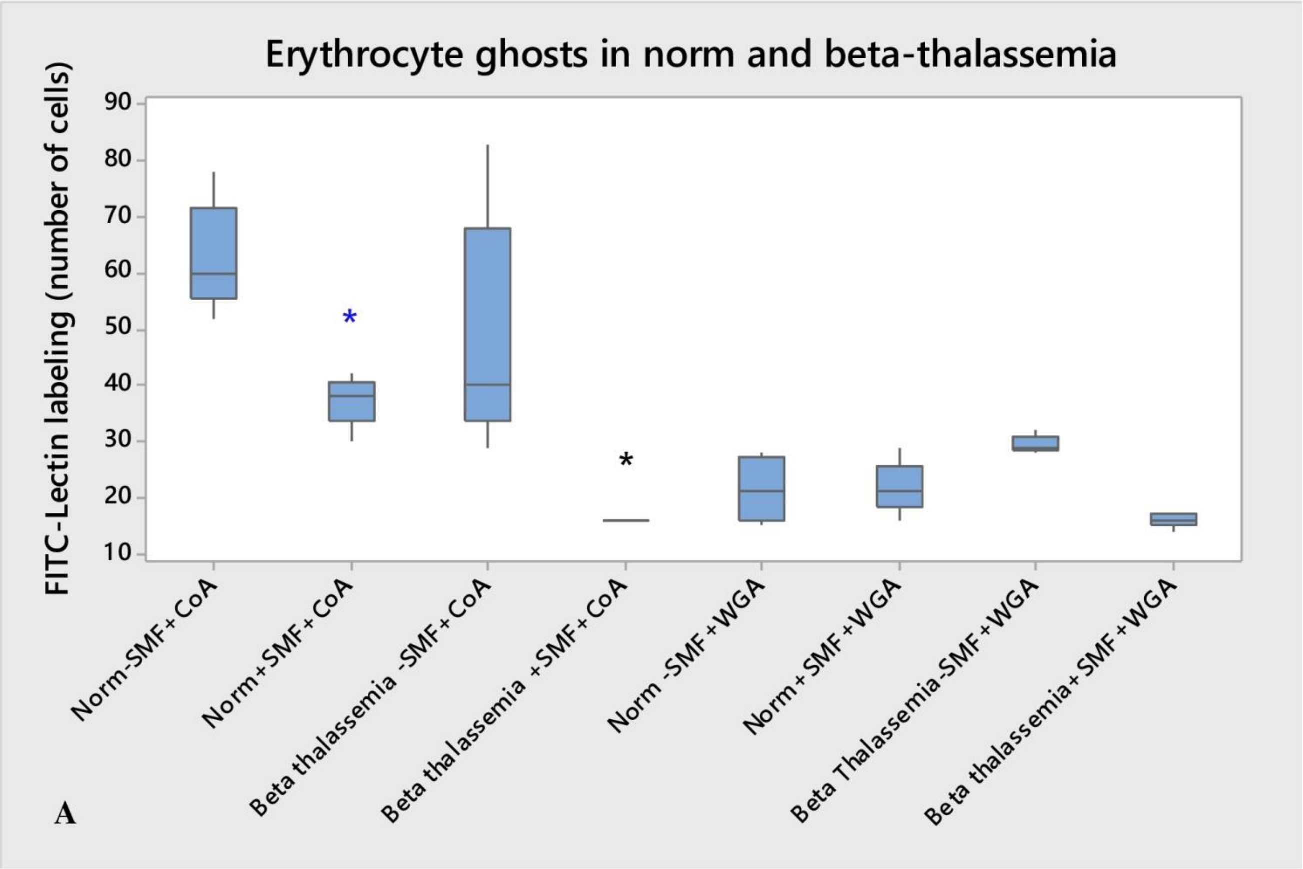

Erythrocyte ghosts in norm and beta-thalassemia
FITC-Lectin labeling (number of cells)
90
80
70
60
50
40
30
20
10
Norm-SMF+CoA
Norm+SMF+CoA
Beta thalassemia -SMF+CoA
Beta thalassemia +SMF+CoA
Norm -SMF+WGA
Norm+SMF+WGA
Beta Thalassemia-SMF+WGA
Beta thalassemia+SMF+WGA
A

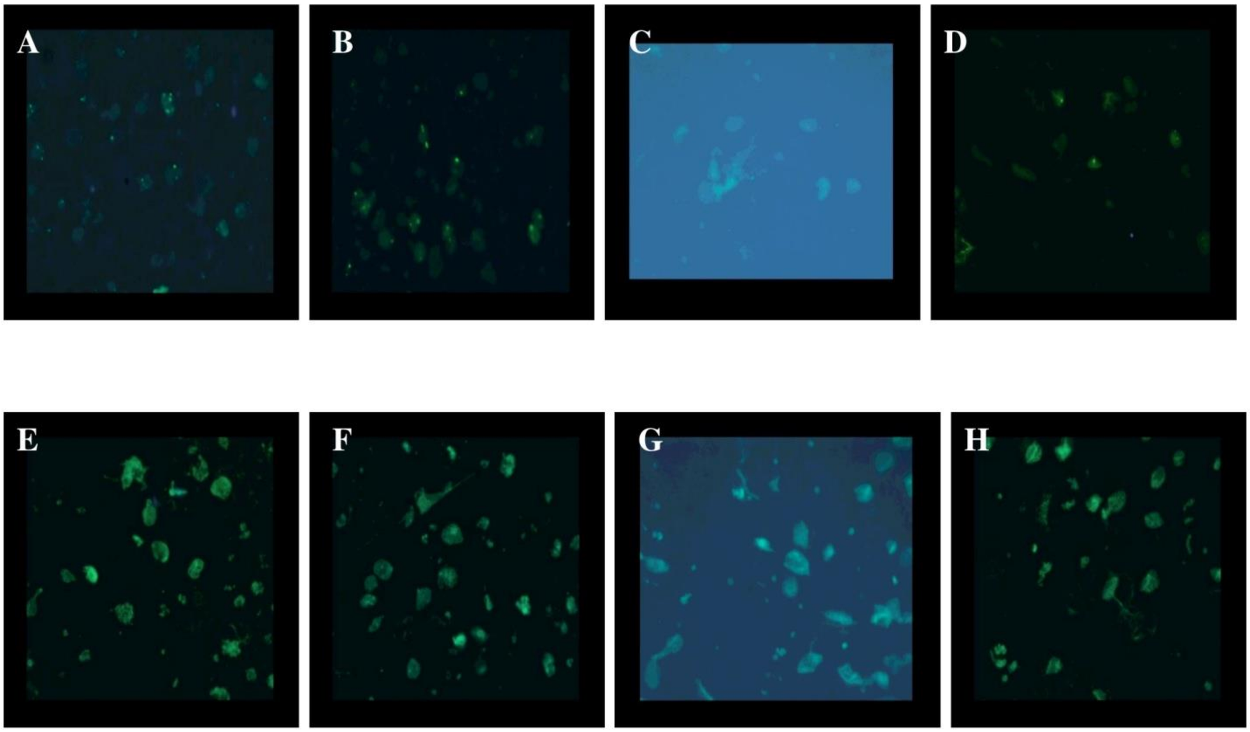

B

Supplementary Figure 8 (A) FITC-lectin labeling of erythrocyte membranes (erythrocyte ghosts) in norm and β-thalassemia after SMF (2.0 T) treatment. FITC-concanavalin A (FITC-CoA) and FITC-wheat germ agglutinin (FITC-WGA) labeling of erythrocyte membranes in norm and β-thalassemia after SMF (2.0 T) treatment. The One-Way ANOVA for norm- and pathology data are performed. The differences among the means are significant (* $p < 0.05$). Each value is the mean ± SD of three independent preparations with three repetitions each. * $p<0.05$ compared to FITC-CoA-containing control in norm (blue asterisk) or FITC-CoA-containing control in β-thalassemia (black asterisk).

(**B)** Examples of FITC-Lectin labeling of human erythrocytes ghosts from healthy subjects and β-thalassemia patients upon SMF (2.0 T) exposure. (**A**) Effect of FITC-concanavalin A (FITC-CoA) on erythrocytes ghosts from healthy subjects without SMF (2.0 T) treatment. (**B**) Effect of FITC-concanavalin A (FITC-CoA) on erythrocytes ghosts from healthy subjects after SMF (2.0 T) treatment. (**C**) Effect of FITC-concanavalin A (FITC-CoA) on erythrocytes ghosts from β-thalassemia without SMF (2.0 T) treatment. (**D**) Effect of FITC-concanavalin A (FITC-CoA) on erythrocytes ghosts from β-thalassemia upon SMF (2.0 T) treatment. (**E**) Effect of FITC-wheat germ agglutinin (FITC-WGA) on erythrocyte ghosts from healthy subjects before SMF (2.0 T) treatment. (**F**) Effect of FITC-wheat germ agglutinin (FITC-WGA) on erythrocytes ghosts from healthy subjects after SMF (2.0 T) exposure. (**G**) Effect of FITC-wheat germ agglutinin (FITC-WGA) on erythrocyte

ghosts from β-thalassemia patients before SMF (2.0 T) treatment. (**H**) Effect of FITC-wheat germ agglutinin (FITC-WGA) on erythrocyte ghosts from β-thalassemia patients after SMF (2.0 T) exposure.
